# Mind the Gap: A Nationwide Analysis of Case Distribution, Resident Exposure and Institutional Variation in German Pediatric Surgery Training

**DOI:** 10.3390/children13040554

**Published:** 2026-04-16

**Authors:** Sabine Drossard, Maria Christina Stefanescu, Andrea Schmedding

**Affiliations:** 1 Department of General, Visceral, Transplant, Vascular and Pediatric Surgery, University Hospital Würzburg, 97080 Würzburg, Germany; 2 Department of Pediatric Surgery, University Medical Center of the Johannes Gutenberg University Mainz, 55131 Mainz, Germany; 3 Department of Pediatric Surgery and Pediatric Urology, City Hospital Braunschweig, 38124 Braunschweig, Germany

**Keywords:** surgical training, surgical education, volume-outcome, medical education, pediatric surgery, residency, operative exposure, workforce, caseload

## Abstract

**Highlights:**

**What are the main findings?**

• This German nationwide analysis (2012–2023) mapping OPS codes to training categories in pediatric surgery shows that training-category coverage varies by hospital type and trended downward over time, with the greatest strain in very low-frequency domains.
• Higher surgical volume correlates with broader category coverage and more residents, but rare procedures remain difficult to distribute evenly across training sites.

**What are the implications of the main findings?**

• Pediatric surgical training capacity in Germany faces structural challenges, leading to risk of delayed qualification and uneven access to specialized care for children. The results highlight structural challenges and underscore the need to organize resident exposure to low-frequency procedures.
• Transparent national documentation, coordinated inter-hospital rotation networks, and guidance on designated centers for rare procedures are needed to align training with service needs and support workforce stability.

**Abstract:**

**Background**: Pediatric surgical care in Germany is delivered within a highly decentralized system, and training structures vary considerably between institutions. Adequate operative exposure is essential for competency-based training. The specialty requires a high number of operative procedures during training, yet concerns have been raised that residents may not achieve the required case numbers within the standard training period. The German Model Training Regulations (*Musterweiterbildungsordnung*, MWBO) define 22 procedural categories with specific case number targets for pediatric surgery. However, the extent to which current training structures allow for the fulfillment of these requirements remains unclear. This study examines the distribution of procedures and residents across different hospital types and estimates whether the available procedural volume may be sufficient under simplified allocation assumptions. **Methods**: We conducted a nationwide analysis of pediatric surgical training capacity in Germany using procedural data from hospital quality reports published by the Federal Joint Committee (*Gemeinsamer Bundesausschuss*, G-BA) between 2012 and 2023. A total of 3440 OPS codes were assigned to 22 training categories, and case volumes were analyzed across different hospital types. The estimated training capacity was calculated assuming even distribution of cases among residents, and that all eligible procedures are performed with full resident access. **Results**: Data from an average of 82.3 pediatric surgical departments per year were analyzed, including 29.7% university hospitals, 58.7% non-university departments, and 11.7% other institutions. Most departments reported fewer than five residents. Between 2012 and 2023, the mean number of residents increased slightly across all hospital types, while inpatient numbers declined. Consequently, inpatient exposure decreased from 469.8 to 354.0 cases per resident per year. Patient exposure differed significantly by institutional category (*p* < 0.001), with higher exposure in non-university departments compared with university hospitals. Across all hospital types, the mean number of fulfilled procedural training categories declined over time. No institution met the target numbers for all categories without cooperation with other units. Thoracic surgery procedures were least frequently covered, whereas appendectomies and inguinal hernia repairs were most consistently fulfilled. Distinct patterns of subspecialization emerged, with trauma procedures less frequently reported at university hospitals and thoracic procedures less frequently reported at non-university departments. Although the overall national procedural volume appears sufficient for most training requirements, low-volume and highly specialized procedures were concentrated at selected centers, limiting their accessibility for trainees. **Conclusions**: Even though there are sufficient pediatric surgical procedures in Germany, they are unevenly distributed between hospitals. Under a simplified allocation model, many pediatric surgical departments in Germany currently lack sufficient procedural volume to meet training requirements in the defined training timeframe for all trainees. Structural reforms—including mandatory national documentation, minor MWBO adjustments and the creation of training networks—are necessary to ensure comprehensive and equitable pediatric surgical education. Without these changes, extended training durations and reduced trainee satisfaction may contribute to workforce shortages and limit the future quality of pediatric surgical care in Germany.

## 1. Introduction

Pediatric surgery is one of the most diverse surgical specialties in Germany, encompassing abdominal and thoracic surgery, oncological surgery, traumatology, burn medicine, plastic surgery, and urology, as well as procedures involving the head and neck. Additionally, pediatric surgeons require knowledge in neurosurgery and vascular surgery [1]. Beyond common procedures such as appendectomies and inguinal hernia repairs, the treatment of congenital and acquired rare and complex diseases is also an integral part of the field.

In addition to technical skills, residency training is fundamental in fostering core surgical ethics and professional identity formation. Surgeons must learn to assume responsibility for vulnerable patients, act with integrity within hierarchical team structures, and make complex decisions under conditions of uncertainty. These principles are primarily acquired through clinical training and mentorship and shape long-term professional behavior [2]. They are essential not only for safe patient care but also for the development of a sustainable and responsible surgical career. The development of these competencies depends on adequate training structures and clinical exposure.

Pediatric surgery in Germany has historically developed in a largely unregulated manner. As in many other developed countries worldwide, individual surgeons, gynecologists, and pediatricians began to specialize in the surgical care of children in the early 19th century [3,4]. In Germany the development started in 1815, the first children’s hospitals were established from 1859 on. Until 1962, about 20 departments of pediatric surgery were founded. From 1963 onward, there was a rapid increase in pediatric surgical departments [5,6]. In East Germany, this expansion was centrally directed by the state, whereas in West Germany it occurred without centralized planning [7,8]. By 1984, a total of 89 pediatric surgical departments had been established. Between 1985 and 2004, the number grew only slowly to 93 departments [9]. From 2005 onwards, a policy decision mandating that every highest-level perinatal center must be supported by pediatric surgery [10] led to the establishment of new, smaller pediatric surgical units and to the relocation of existing sites [4].

As a result of this historical development in Germany, pediatric surgical care is delivered in a decentralized system [11]. Over 130 hospital departments [12], which belong to 7% of all hospitals [13], along with roughly 100 private practices and medical care centers [12], provide surgical services performed by board-certified pediatric surgeons. In 2023 there were 538 board-certified pediatric surgeons working in hospitals (0.64 per 100,000 population) and 140 working in private practices (0.17 per 100,000 population) [14]. Regional analysis reveals that almost all regions with over 100 children per square kilometer have a hospital offering pediatric surgery [9].

A further consequence of unregulated development is, that no single department covers the full scope of the specialty [9]. Since there are no mandatory requirements for children to be treated by specialized personnel, surgeons without formal training in pediatric surgery also perform operative procedures on children. Because community and non-profit hospitals developed pediatric surgical units earlier than universities [4], pediatric surgical departments at university hospitals are often smaller than those at community or dedicated children’s hospitals [15], a situation that contrasts with many other countries. Notably, two of the largest university hospital departments (Mannheim and Augsburg) originated as former community hospitals.

Formal training in pediatric surgery was first established in 1955 in East Germany. It was suspended in 1967 and re-established in 1974 [8]. In West Germany, a subspecialty training program was introduced in 1968, which could be pursued after certification as a specialist in general surgery. Following German reunification in 1990, a board certification in pediatric surgery was introduced in 1992, which could be obtained without prior specialization in general surgery [7]. Since then, the defined minimal training duration comprises six years. The last reform of pediatric surgery specialty training in 2018 included six months in emergency care and six months in intensive care of children and adolescents within this timeframe [16].

In Germany, the number of residency positions in pediatric surgery is not determined through centralized workforce planning but is defined locally by individual hospitals according to their staffing structures, service demands, and financial resources. Department heads apply to the State Medical Chamber for accreditation to provide training, while residents apply directly to pediatric surgical departments. Therefore, national data on application numbers and demand for pediatric surgery training positions are not systematically available.

Surgical training in Germany remains largely unstructured [17], which also applies to pediatric surgery, where training is influenced by institutional factors and shows considerable variability in quality and operative exposure [18]. The training environment varies substantially between hospitals in terms of size, case volume and diversity, and the number of medical trainees and specialists [9,19]. Data on the number of residents, training duration, and overall quality remain limited. As of 2024, 116 pediatric surgeons in Germany are accredited to provide training in pediatric surgery, with an average of 40 residents achieving board certification annually [18].

As the number of residents per department is not standardized, off-hour care models vary accordingly. In well-staffed pediatric surgery centers, residents in pediatric surgery typically provide initial patient care during off-hours. In contrast, institutions with fewer pediatric surgery residents rely on a pooled on-call system, where initial care is delivered by a team comprising residents from various specialties, such as pediatrics, general surgery, urology, and pediatric surgery. Depending on the on-call model, residents usually complete between four and eight on-call shifts per month.

By the end of their training, pediatric surgeons should be proficient in both routine and emergency procedures and possess comprehensive expertise in the care of children and adolescents with surgical conditions. The Model Training Regulations (*Musterweiterbildungsordnung*, *MWBO*) specify 22 procedural categories for Pediatric Surgery, which are associated with defined target numbers; two procedures provide a sub-division for procedures performed in infants. Training in pediatric surgery requires a minimum of 410 operations for board eligibility, more than trauma surgery (375) or cardiac surgery (270), reflecting a high procedural demand [16].

A recent survey among German pediatric surgery trainees indicates that many pediatric surgery residents are unable to complete their training within the standard timeframe, primarily due to challenges in meeting the required minimum number of surgical procedures [18]. Insufficient operative exposure is widely perceived by surgical residents as a major training problem [18,20,21,22] and is linked to trainee dissatisfaction [20,23,24]. Therefore, the discipline may be particularly sensitive to constraints in case availability.

This study examines whether there are sufficient procedures available for all residents, how the number of training procedures and residents varies across different department types, and how procedures and residents are distributed according to different hospital levels. The overarching goal is to inform workforce planning and shape future training policies.

## 2. Materials and Methods

All hospitals, which provided pediatric surgical services, according to the German Society of Pediatric Surgery [12], were defined. The pediatric surgical departments were divided into three groups: those at university hospitals, non-university departments, and other units, which were sub-units of pediatric or surgical departments or small units (Supplementary Figure S1).

To assess the structural conditions of pediatric surgical training, hospital quality reports published annually by the Federal Joint Committee (Gemeinsamer Bundesausschuss, G-BA) between 2012 and 2023 were analyzed. These reports include data on the total number of in-house patients, the number of operative procedures (both inpatient and outpatient), and staffing details, such as the number of surgical personnel and board-certified pediatric surgeons per department. The analysis was conducted using publicly available aggregated data; no patient-level data were accessed. All pediatric surgery units that reported their procedural and personal data were included in the analysis. Units in which pediatric surgery was reported in aggregate with pediatric or adult surgical services were excluded from analysis, as pediatric procedures and staffing were not disaggregable.

Staff count was provided by the quality reports as full-time equivalent (FTE). We defined patient exposure per department and year as total number of patients divided by sum of FTE. Where the reported cumulative resident count was <1.0 full-time equivalent (FTE), we imputed 1.0 FTE to avoid inflating per-resident exposure. According to the number of in-house patients treated departments were divided into three groups: High-volume units = >2000 patients/year, mid-volume units = 1000 to <2000 cases per year, low-volume units = <1000 cases per year.

MWBO-defined procedures were matched with codes of the Procedure Classification Catalogue (*Operations-und Prozedurenschlüssel*, OPS) as follows: We first generated a comprehensive list of all procedures reported in the hospital quality reports and extracted the corresponding OPS codes. These codes were then systematically mapped to the MWBO-defined pediatric surgery categories (Table 1) or flagged as “not included” if no match was possible. For procedures comprising multiple components, only the primary procedure was retained for analysis to avoid double counting. In total, 3440 of 16,414 OPS codes were assigned to the predefined training categories. Because the quality reports do not distinguish patients’ age, infant-specific analyses were not feasible. Category definitions varied in code specificity: some categories mapped to distinct, specific OPS codes, whereas others required grouping of several less-specific codes. OPS code matching and inclusion were performed independently by three researchers. Any discrepancies were discussed, and consensus was reached in all cases. The full code list is provided in Supplementary Table S1. For secondary analyses, we defined a subset of procedures as “Basic Surgery,” i.e., operations widely regarded as training cases and routinely performed by residents in the early years of training (Table 1). Inclusion criteria were discussed by the research team, and consensus was achieved.

For the purposes of this study, the effective training period in pediatric surgery was set to five years to account for mandatory non-surgical rotations within the nominal six-year program. To estimate training adequacy, we calculated, for each department and MWBO category, the number of procedures per resident and year. These numbers were compared with the MWBO target numbers per year (equal to target numbers divided by 5). This comparison was done under the assumption that all eligible procedures are performed by residents and procedural opportunities are evenly distributed among residents in that department. To further assess procedural availability, we estimated the number of years required for the cumulative cohort of residents to fulfill the minimum procedural requirements specified by the training regulations, assuming the total number of procedures performed annually in Germany. This calculation allowed us to approximate whether the national procedural volume is sufficient to meet training demands.

### Statistical Analysis

Further statistical analyses were performed for patient exposure and fulfillment of training categories.

Patient exposure was defined as the number of patients treated per full-time equivalent (FTE) residents:

$$Patient\;exposure=\frac{total\;number\;of\;patients}{FTE\;residents}$$

This represents a continuous rate rather than a count or proportion. Because the number of trainees is measured in FTE (including fractional values, e.g., 0.5), the resulting outcome is continuous and not restricted to integer values.

Given the continuous nature of the outcome, count-based models (Poisson or negative binomial) or beta regression were not appropriate. We therefore used linear mixed-effects models (LMMs), which allow for continuous outcomes and inclusion of random effects to account for clustering at the hospital level. Due to right-skewness, a log-transformation was applied, which improved normality. The variable “year” was centered at the baseline year to facilitate interpretation: the model intercept represents the expected patient exposure in the reference group at baseline. Fixed effects included hospital type, year, and their interaction, and pairwise comparisons were performed using estimated marginal means with Tukey correction.

Fulfillment of training categories was analyzed at the individual procedure level. A linear mixed-effects model was first fitted using the number of fulfilled categories per clinic-year as the outcome:

number fulfilled ~ HospitalCategory+(1|DepartmentID)

To account for potential temporal trends, calendar year was added as a fixed effect in a secondary model. Random intercepts for clinics accounted for repeated measurements.

As a sensitivity analysis, a binomial mixed-effects model was fitted at the procedure level, modeling the probability of fulfilling a category (1 = fulfilled, 0 = not fulfilled) with random intercepts for each clinic:

fulfilled ~ HospitalCategory+ Year+(1|DepartmentID)

Odds ratios and 95% confidence intervals were derived from model estimates. This approach allowed us to account for clustering and variability between clinics while evaluating temporal trends and category-specific differences.

Analyses were performed using available data only. If data for a given clinic-year were missing, those observations were excluded from the relevant analysis. Statistical significance was defined as *p* < 0.05.

Data were managed and processed using Microsoft Access^®^ and Microsoft Excel^®^ (Microsoft Office Professional Plus 2019, Microsoft Corporation, Redmond, WA, USA). Statistical analyses were performed using R, Version 4.5.2 (R Foundation for Statistical Computing, Vienna, Austria). Programming support from ChatGPT-5.2 (OpenAI, 13 February 2026 version) was used solely for R code drafting and troubleshooting. All code and results were independently verified, adapted, and reproducibly executed by the authors.

## 3. Results

### 3.1. Structure

**Figure 1:**
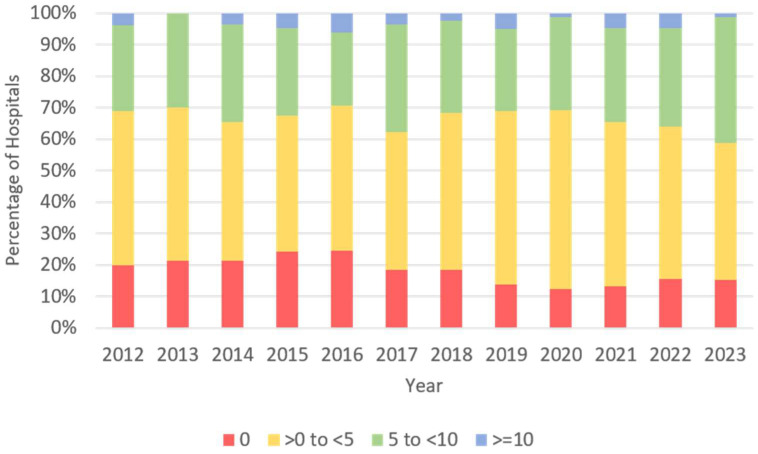
Distribution of residents per department grouped in 4 categories (0 = no residents, <5 = 1–4 residents, <10 = 5–9 residents, >=10 residents) displayed as stacked bars indicating the percentage of hospitals in each category.

On average, data from 82.3 departments were available per year, with 29.7% representing university hospitals, 58.7% non-university departments, and 11.7% other institutions (Supplementary Figure S1). The institutions treated a mean number of in-house patients per year of 1203.4 in university hospitals, 1613.8 in non-university departments, and 378.5 in other institutions. On average, 81.2% of hospitals had at least one resident. Across all years, most departments reported fewer than five residents, whereas departments with 5–9 residents accounted for approximately one quarter to one third of sites. Departments with ≥10 residents remained uncommon. (Figure 1) Detailed analysis of the structural parameters regarding hospital types is provided in Table 2.

### 3.2. Patient Exposure

**Figure 2:**
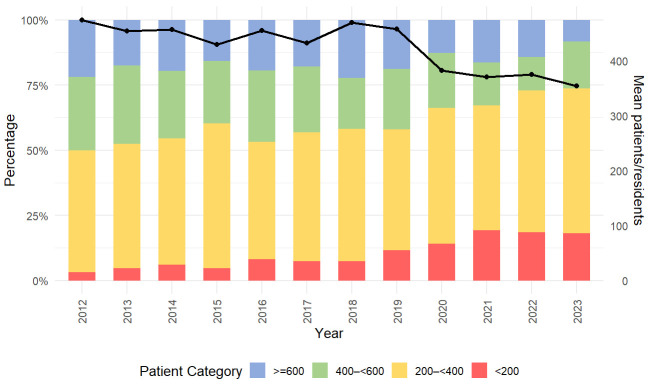
Distribution of caseload and number of patients per resident and year. Percentage of hospitals with defined patient exposure categories depicted as stacked bars. Mean number of patients per resident depicted as black graph.

Patient exposure per resident was assessed based on inpatients only, as data were available for ambulatory procedures but not for ambulatory patients. Among departments with residents, the mean number of inpatients per resident declined from 469.8 in 2012 to 354.0 in 2023, with the most pronounced decrease occurring during the COVID-19 pandemic (Figure 2).

In the linear mixed-effects model with log-transformed patient exposure, both clinic type and year were significantly associated with patient exposure (*p* < 0.001). In the reference group (university hospitals at baseline year), the estimated patient exposure was approximately 305 patients per trainee. Compared with university hospitals, non-university clinics had a 65% higher patient exposure (β = 0.503, *p* < 0.001), while other institutions showed a non-significant 31% increase (β = 0.270, *p* = 0.10). Over time, patient exposure decreased significantly by approximately 2.6% per year (β = −0.027, *p* < 0.001). There was substantial variability between clinics, supporting the inclusion of random intercepts.

### 3.3. Training Procedures

**Figure 3:**
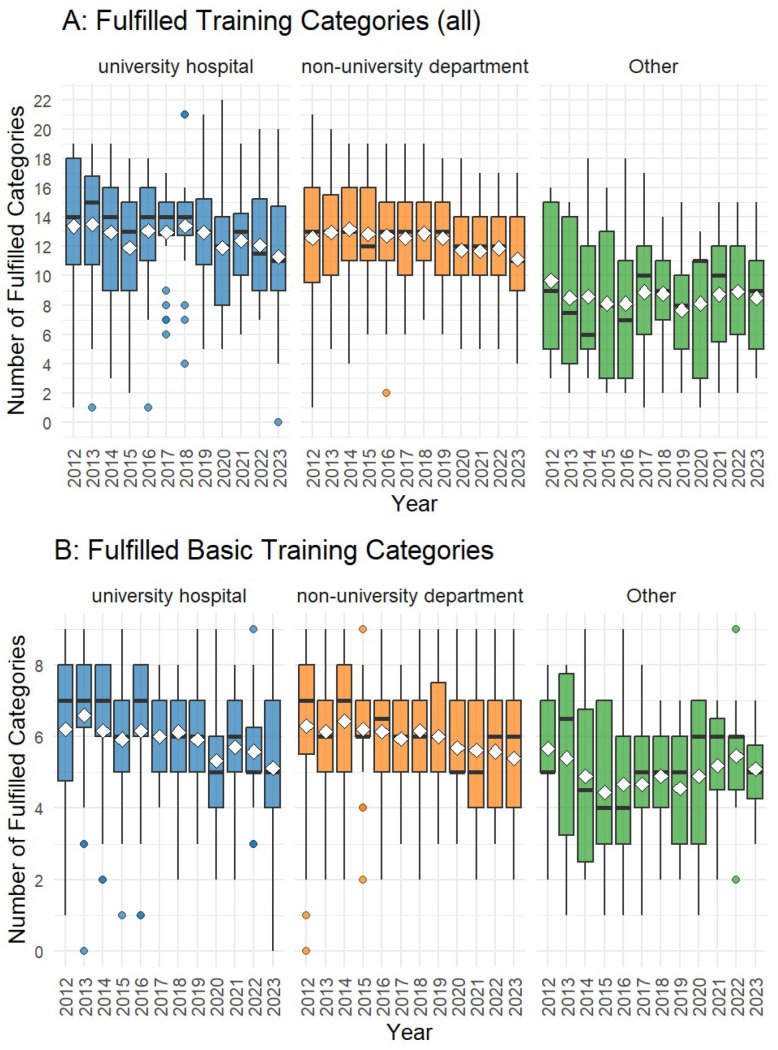
Number of fulfilled training categories per year and kind of hospitals depicted as boxplots. Black line = Median, white diamond = Mean, dots = outliers. (**A**): All categories (max: 22). (**B**): Basic training categories (max. 9). For each department, the annual number of procedures performed within a given category was divided by the number of residents in that department during the same year. For departments with less than one resident, the number of residents was set to one. A category was considered fulfilled if the resulting value exceeded the reference number of procedures for that category divided by five.

Over the study period, the mean number of fulfilled procedural categories declined across all hospital types. In university hospitals, the average decreased from 13.38 in 2012 to 11.31 in 2023; in non-university departments, from 12.60 to 11.12; and in other hospitals, from 9.33 to 8.50 (Figure 3). In 2020, one university hospital fulfilled all training categories; however, this was attributable to the inclusion of data from the traumatology department, which provided training in pediatric traumatology.

Compared to university hospitals, other institutions fulfilled significantly fewer categories (β = −3.40, *p* < 0.001), corresponding to approximately three fewer fulfilled categories out of 22. No significant difference was observed between non-university departments and university hospitals.

Calendar year had a significant overall effect (*p* < 0.001, LMM). While fulfillment remained relatively stable from 2013 to 2019, a significant decline occurred from 2020 onwards. In particular, 2020 (β = −1.10, *p* = 0.007), 2021 (β = −0.97, *p* = 0.017), 2022 (β = −1.00, *p* = 0.014), and 2023 (β = −1.49, *p* < 0.001) showed statistically significant decreases compared to 2012. This indicates a non-linear temporal trend with a marked reduction in recent years.

Considering the nine core surgical procedures defined as basic surgery, six institutions (three university hospitals and three non-university hospitals) fulfilled all training requirements in 2012, whereas in 2023 only one university hospital and two non-university hospitals met these thresholds. For basic training categories, no significant differences in fulfillment were observed across hospital types.

### 3.4. Specialization of Departments

**Figure 4:**
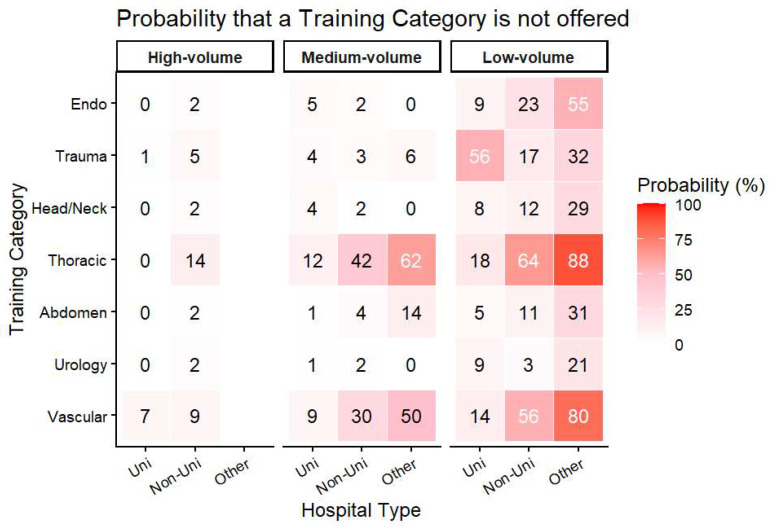
Probability that a training category is not offered at all (in percent), stratified by hospital type and size. High-volume units = >2000 patients/year, mid-volume units = 1000 to <2000 cases per year, low-volume units = <1000 cases per year.

**Figure 5:**
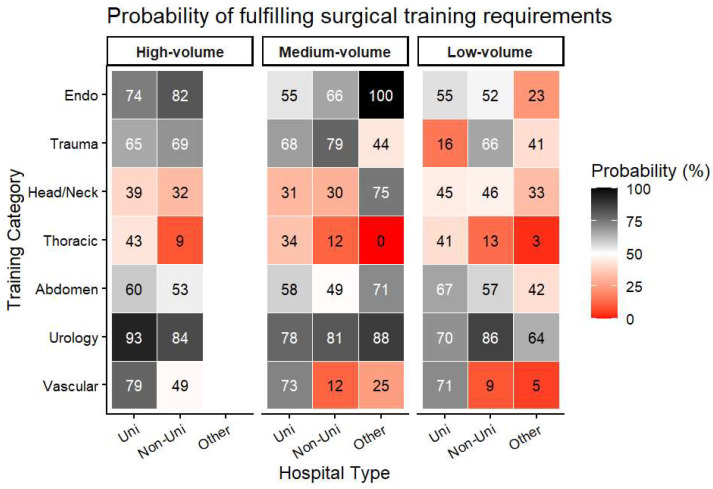
Probabilities of fulfilling training requirements by hospital type and hospital size. Uni = university hospitals; non-uni = non-university departments; other = other departments.

Different areas of specialization in specialist medical training emerged at university and non-university hospitals. Some MWBO categories were not reported at all by a sizeable proportion of units. In university departments, this was most evident for trauma procedures, in non-university departments for thoracic surgery (Figure 4 and Figure 5). Among all categories, appendectomies, cryptorchidism procedures, and inguinal hernia repairs were the most consistently fulfilled.

### 3.5. Total Number of Procedures and Residents

The mean cumulative number of residents per year was 106.5 at university hospitals, 187.6 at non-university departments, and 2.9 at other departments. For most procedural categories, the total number of procedures performed in Germany are sufficient to support adequate training opportunities for all trainees (Table 3). However, while commonly performed procedures were generally available in adequate numbers, lower-volume and highly specialized procedures were disproportionately concentrated at selected centers, thereby limiting their accessibility for all trainees (Table 3).

## 4. Discussion

This study demonstrates that many pediatric surgical departments in Germany do not provide sufficient procedural volume across all MWBO-defined categories to enable trainees to complete residency within the standard timeframe at a single institution. Notably, no institution met the full training requirements in any analyzed year, even under idealized assumptions.

Pediatric inpatient case numbers in Germany have declined by 11% in absolute terms and by 16% per 100,000 children following the COVID-19 pandemic [25]. This is consistent with our findings, which also show a decline in high-volume units alongside an increase in medium- and low-volume institutions. Changes in working conditions, driven by European Union legislation [26] and collective agreements of the *Marburger Bund* (German Trade Union of Employed Physicians) [27], have reduced weekly working hours and on-call duties while increasing the number of residents per department. Together, these factors have likely contributed to the decrease in patient exposure observed in our study.

While our study assumes that all eligible procedures are performed with full resident participation, evidence from other surgical specialties in Germany suggests that operative training is limited not only by case numbers but also by how procedures are allocated to residents. Huber et al. reported that only 24.4% of index procedures in German surgical residency were performed entirely by residents, whereas laparoscopic cholecystectomy, considered a basic procedure, was completed by residents in 43.3% of cases [28]. Similarly, a 2018 survey of German urology residents found that surgical exposure was generally low and largely restricted to minor and intermediate-complexity procedures [29]. These findings indicate that institutional case volume does not directly translate into resident experience. Rather, resident participation appears to be substantially higher for routine procedures than for complex operations. In the context of our results, this implies that our estimates likely represent a best-case scenario and that actual training opportunities, particularly for rare or complex procedures, may be even more limited.

Evidence from general surgery residency programs in the United States shows a paradoxical trend: although total case numbers per resident have increased, procedural diversity has declined over time [30,31]. This narrowing of operative experience raises concerns about preparedness for independent practice. Declining caseloads have also been reported among board-certified pediatric surgeons in the United States [32], raising concerns not only for training but also for maintaining proficiency in rare, high-stakes procedures. Limited exposure to index cases among practicing surgeons highlights the need for structured approaches to procedural competency throughout both training and professional practice. Collaborative models, such as centralized hubs for complex procedures, may offer promising solutions [33].

Internationally, pediatric surgery training is structured in highly diverse ways. A 2024 study covering 44 countries revealed that, in many cases, national training programs in pediatric surgery do not adequately reflect the actual demand for pediatric surgeons [34]. In several high-income countries, such as the UK, the US, and Australia/New Zealand, training is embedded in structured competency-based systems with defined supervision levels, formal assessments, and nationally standardized training requirements. [35,36] In Europe, the Union of European Medical Specialists (UEMS) has a statutory purpose to harmonize and improve the quality of medical specialist practice across the European Union [37]. After showing substantial differences between the European Countries [38], the European Syllabus in Paediatric Surgery was published in 2009 [39]; however, eight years later, these substantial differences between European countries were still documented [40]. Whether the transition from a syllabus to the European Training Requirements, published in 2020 [41], will lead to an improved and more comparable training remains to be determined. In contrast to high-income countries, the literature from low- and middle-income countries highlights a different challenge: limited access to formal training programs and persistent workforce shortages, with some countries relying on shorter or direct-entry pathways to expand pediatric surgical capacity [42].

Research indicates a positive correlation between surgical case volume and training efficacy [43,44,45], emphasizing the importance of progressive responsibility in surgical education, where residents initially perform basic procedures before advancing to more complex operations [46]. Furthermore, operative volume is the most commonly cited quality indicator for surgical training [47]. Exposure to a broad procedural spectrum strengthens diagnostic reasoning and clinical decision-making, contributing to overall surgical competence [46]. While the association between surgical case volume and surgical learning curves has been shown across different adult surgical specialties, corresponding data in pediatric surgery remain limited [48]. Robust evidence connecting individual trainee case volume with competency acquisition or clinical outcomes is still lacking.

Insufficient procedural exposure during residency jeopardizes the development of surgical competence and may negatively affects trainees [20]. As a consequence, training duration may be prolonged, which has been associated with reduced resident satisfaction [49], increased stress, and higher attrition rates [50,51], all of which pose a threat to the long-term sustainability of the pediatric surgical workforce. This is especially concerning considering anticipated future shortages of qualified surgeons. Extended training durations, which mainly result from limited operative exposure [18], may disproportionately affect female trainees, who face higher rates of delayed childbearing, infertility, and pregnancy-related complications [52].

These findings suggest that insufficient operative exposure is not only a technical training issue but has broader implications for well-being, career trajectories, and workforce stability. Key determinants of trainee satisfaction include strong clinical supervision, an appropriate workload, and a supportive training environment [53]. Although data on the prevalence of professional dissatisfaction among pediatric surgeons in Germany are scarce, available evidence points toward substantial structural stressors, including high work–family conflict and limited perceived ability to balance professional and personal responsibilities [54]. In addition, dissatisfaction with training conditions has been reported among German pediatric surgery trainees, including changes in training sites due to inadequate training environments [18]. These findings are consistent with broader surgical literature, where reduced operative autonomy in trainees has been associated with burnout and professional dissatisfaction [55].

However, at the national level, sufficient pediatric surgical case volumes are available for training in most categories. Training deficits therefore appear to arise primarily from unequal case distribution and rigid procedural requirements rather than from an overall shortage of cases. Exposure gaps vary by institutional setting: trauma procedures are frequently absent in university departments, whereas thoracic surgery and central venous access are less available in non-university units. These patterns reflect service specialization within the German healthcare system; for example, central venous access is concentrated in hospitals with pediatric oncology services, typically university centers.

This mismatch between training requirements and structural realities underscores the need for structured training networks, mandatory rotations, and national monitoring systems to ensure equitable operative exposure [56]. While previous studies have reported improved surgical outcomes when procedures are performed by specialized pediatric surgeons compared to non-specialists [57,58], our study did not assess outcome differences between provider groups. Nevertheless, pediatric surgical procedures may still be performed by surgeons without formal pediatric training.

Within the German healthcare system, comparatively lower reimbursement for pediatric cases, may incentivize hospitals to manage these patients within general surgical departments rather than invest in dedicated pediatric surgical services, as seen in pediatrics [59]. This economic dynamic risks progressively shifting case volumes away from pediatric surgery. Such a development would not only affect care structures but is also likely to erode the procedural basis required for high-quality pediatric surgical training. At the same time, broader system-level changes, including hospital restructuring and the expansion of ambulatory surgery, are expected to further reduce inpatient case volumes in pediatric surgery [56]. Without deliberate countermeasures, these trends may substantially compromise training opportunities. Addressing this challenge will likely require the systematic integration of smaller units, outpatient clinics, and office-based settings into formalized training pathways.

Against this background, care pathways for pediatric patients should be designed to ensure access to appropriately trained pediatric surgical expertise, particularly for rare and complex conditions, while simultaneously safeguarding sufficient case exposure to maintain training standards.

As our data suggest, adaption of training categories could be beneficial in some cases. The categories of head and neck procedures appear overly narrow and may benefit from expansion to better reflect clinical practice. From a training perspective, the competencies required are largely comparable to those for excisions at other anatomical sites.

In response to decreasing availability of index cases, simulation-based training and surgical skills workshops provide valuable supplementary learning opportunities and preparation for rare but critical procedures [60]. Although increasingly adopted and well suited for complex skill acquisition, simulation has not yet been consistently integrated into curricula or mandated across German residency programs [61]. Recent efforts to expand online educational formats have further enhanced access to theoretical and case-based learning across geographic boundaries [62]. In addition, a stepwise approach to gradually increase operative responsibility should be integrated into training curricula to promote progressive autonomy while ensuring patient safety, as proposed by other surgical disciplines [28,63,64].

Nevertheless, hands-on operative experience remains crucial for pediatric surgical training. Direct participation in surgical procedures enables the development of technical skills, clinical judgment, and confidence in the operating room. Exposure to diverse cases fosters decision-making, situational awareness, and the ability to manage intraoperative complications—competencies essential for independent surgical practice.

### 4.1. Recommendations

(1) In the short term, minor MWBO adjustments would improve alignment: (i) reducing the assistance in thoracotomy target to 10 procedures; (ii) merge head-and-neck subcategories by omitting the separate branchial cleft/cyst requirement and subsuming all head-and-neck procedures under one category together with other procedures of the body’s surface.
(2) To ensure comprehensive training, structured training networks should be established [56], facilitating mandatory rotations between different hospitals and outpatient settings to enhance trainees’ surgical exposure and competency development.
(3) All national procedure databases should include information on the medical specialties performing these procedures in order to provide stronger evidence base for planning pediatric surgical care and training.
(4) In the long term, fundamental reforms are needed to strengthen surgical education and align training needs with clinical realities [65]. A crucial first step toward improving surgical training would be nationwide, mandatory documentation tracking all pediatric surgery trainees and their procedural experience. Such a system would allow for transparent monitoring, facilitate benchmarking across institutions, and help identify structural gaps in training opportunities.

### 4.2. Strengths and Limitations

This study is the first to provide a nationwide, longitudinal analysis of procedural volumes and structural conditions relevant to pediatric surgical training in Germany. It presents model-based estimates of training opportunities rather than proven fulfillment of training requirements. Currently, no structured national system exists for tracking training progress or procedural experience in pediatric surgery.

Several limitations should be considered. First, some units had to be excluded because pediatric surgical data were reported jointly with other surgical services or were incomplete, which may have introduced selection bias. Second, the use of retrospective administrative data is inherently limited by possible coding inaccuracies, reporting inconsistencies, and missing information, e.g., the reported number of procedures for congenital abdominal malformations (cat. 141) likely underestimates the true case volume. This is attributable to the fact that only a limited number of malformations are associated with specific procedural codes, such as abdominal wall defects or anorectal malformations. In contrast, other congenital anomalies, including intestinal atresia, are generally treated with non-specific procedures, such as intestinal anastomosis or stoma formation, which were assigned to other categories in the analysis.

Third, our calculations relied on several assumptions that may have led to over- or underestimation of availability of procedures for training purposes. For example, closed fracture reductions and cast-only treatments in emergency care may not have been coded consistently across hospitals, likely resulting in an underestimation of trauma-related exposure. In addition, we assumed that all eligible procedures were performed by residents and that cases were distributed evenly among them. Both assumptions are unlikely to reflect clinical reality, where operative participation is influenced by procedure type, institutional case allocation, varying levels of experience, shift work, and leave—all of which contribute to unequal access to operative opportunities. Conversely, residents from non-surgical specialties on training rotation (e.g., pediatric trainees) may have been included in staffing counts despite not being required to complete the pediatric surgery procedure catalog, potentially inflating trainee numbers.

Importantly, our analysis uses procedural volume as a surrogate marker of training opportunity and does not assess actual competency acquisition or patient outcomes. The procedural coding system does not differentiate between cases of varying complexity or educational value. Case numbers alone therefore cannot fully reflect the quality or effectiveness of training.

Despite these limitations, the study provides valuable insights into the structural limitations of pediatric surgical education and highlights the need for national documentation systems and better coordination of training exposure across sites.

### 4.3. Recommendations for Future Research

Future research should focus on collecting detailed trainee-level data, including individual case logs, procedural complexity, and the extent of resident involvement in procedures to accurately assess training adequacy and enable benchmarking across institutions. In addition, studies exploring the correlation between operative experience and competency acquisition in pediatric surgery are urgently needed. Longitudinal research should further evaluate how training structures, such as rotations and training networks, affect resident performance, satisfaction, skill development, and career progression.

## 5. Conclusions

This study highlights significant structural limitations in pediatric surgical training in Germany, indicating challenges in residency training that are exemplary for smaller surgical specialties. Germany has sufficient pediatric surgical volume overall; the challenge is its uneven distribution. Many institutions lack the case volume necessary to provide comprehensive training within the standard timeframe. The resulting gaps in operative exposure threaten both trainee development and future workforce sustainability. Furthermore, this study reports model-based estimates of training opportunities rather than proven fulfillment of training requirements, emphasizing the need for systemic reforms. The introduction of training networks seems essential to ensure high-quality, equitable, and future-ready pediatric surgical education. At the same time, surgical training extends beyond the number of procedures performed. The quality of training plays a central role in shaping long-term professional development and may help protect against dissatisfaction despite structural constraints.

## Figures and Tables

**Table 1:** Procedures required for pediatric surgery training with reference numbers as defined by MWBO. No. of Codes = Number of procedural codes used, No. of Proc = Cumulative number of procedures in Germany in 2012–2023. Categories defined as Basic Surgery are marked in gray.

No.	Training Category	Reference No. of Procedures	No. of Codes	No. of Proc. (Inhouse/Outpatient)
100	Gastrointestinal Endoscopies (GI)			
101	Esophago-gastro-duodenoscopy	20	53	31,887/515
102	Rectosigmoidoscopy	10	53	16,501/722
110	Traumatology (Tr)			
111	Conservative Treatment including fracture reduction	50	27	17,437/5691
112	Osteosynthesis of fractures:			
113	-diaphyseal	25	183	33,103/420
114	-meta- and epiphyseal	25	408	59,231/2044
115	Metal implant removal	25	347	21,446/48,977
120	Head and Neck (HN)			
121	Excision of benign tumors, desmoids, ear tags	15	24	7594/509
122	Excison of branchial cleft cysts and fistulas, lymph nodes	10	36	5749/519
130	Thoracic Surgery (Tho)			
131	Thoracotomy as surgical access route, thoracoscopy	10	49	8089/0
132	Thoracic procedures of higher complexity (1st assistance)	15	256	11,339/3
140	Abdomen and Abdominal Wall (Abd)			
141	Correction of congenital malformations (1st assistance)	20	187	10,638/0
142	Procedures in the abdominal cavity, of which	60	423	65,875/489
143	Appendectomy	25	22	66,283/0
144	Laparotomy as surgical access route	15	170	20,414/5
145	Laparotomies of higher complexity (1st assistance)	30	456	7989/3
146	Inguinal herniotomy, of which	75	31	62,731/28,238
147	Herniotomy of abdominal wall hernias	15	61	7963/6851
150	Kidney, Urinary Tract and Genital Organs (Uro)			
151	Basic urologic procedures	50	126	52,377/43,685
152	Orchidopexy and funicolysis	30	20	54,070/15,056
153	Cystoscopy	15	83	44,228/2829
154	Urologic procedures of higher complexity (1st assistance)	20	414	45,872/88
180	Vascular Surgery (Vasc)			
181	Insertion of central venous access systems	10	11	20,215/907

**Table 2:** Structure of analyzed hospitals: number of departments, median annual number of in-house patients, and median annual number of residents. Values for patients and residents are presented as median (minimum–maximum). Units: total number of units, (units with available data). Values are only reported for units with available data as per the inclusion criteria.

Year	University Departments			Non-University Departments			Other		
	Units	Inhouse Patients	Residents	Units	Inhouse Patients	Residents	Units	Inhouse Patients	Residents
2012	37 (24)	1168.5 (93.0/2623.0)	4.5 (0.0/11.5)	47 (47)	1673.0 (365.0/6469.0)	3.0 (0.0/12.0)	43 (9)	476.0 (0.0/1086.0)	0.0 (0.0/2.1)
2013	37 (23)	1114.0 (187.0/2585.0)	4.5 (0.0/10.0)	50 (48)	1504.0 (458.0/4755.0)	3.6 (0.0/9.8)	47 (10)	279.0 (0.0/1077.0)	0.0 (0.0/0.0)
2014	38 (25)	1097.0 (31.0/2607.0)	5.0 (0.0/10.0)	53 (49)	1557.0 (427.0/4533.0)	3.3 (0.0/12.7)	48 (10)	303.0 (0.0/1016.0)	0.0 (0.0/0.2)
2015	38 (25)	1078.0 (58.0/2893.0)	3.6 (0.0/11.2)	53 (49)	1517.0 (377.0/4505.0)	3.7 (0.0/10.7)	49 (9)	366.0 (0.0/939.0)	0.0 (0.0/0.0)
2016	38 (25)	1076.0 (108.0/2492.0)	3.8 (0.0/11.1)	53 (48)	1590.0 (395.0/4752.0)	3.8 (0.0/13.0)	49 (9)	376.0 (78.0/900.0)	0.0 (0.0/2.8)
2017	38 (24)	1088.5 (129.0/2500.0)	4.6 (0.0/10.4)	53 (49)	1538.0 (403.0/4576.0)	3.6 (0.0/14.8)	48 (9)	425.0 (36.0/966.0)	0.0 (0.0/2.0)
2018	39 (24)	1212.0 (101.0/2531.0)	3.9 (0.0/9.4)	54 (49)	1515.0 (384.0/4299.0)	3.8 (0.0/13.0)	47 (9)	418.0 (39.0/750.0)	0.0 (0.0/2.8)
2019	39 (24)	1377.5 (129.0/2780.0)	4.5 (0.2/11.7)	54 (47)	1504.0 (417.0/4013.0)	3.6 (0.0/15.9)	47 (9)	439.0 (48.0/776.0)	0.0 (0.0/3.3)
2020	38 (25)	1135.0 (73.0/2384.0)	4.3 (0.8/9.3)	55 (49)	1290.0 (479.0/3773.0)	3.5 (0.0/13.0)	47 (9)	344.0 (51.0/613.0)	0.0 (0.0/1.1)
2021	38 (24)	1179.5 (85.0/2343.0)	4.5 (0.2/10.2)	55 (49)	1232.0 (446.0/3398.0)	3.9 (0.0/12.9)	50 (11)	330.0 (0.0/552.0)	0.0 (0.0/1.6)
2022	38 (24)	1124.0 (105.0/2396.0)	4.3 (0.0/10.8)	55 (48)	1306.0 (285.0/3135.0)	3.2 (0.0/15.2)	48 (11)	298.0 (49.0/541.0)	0.0 (0.0/1.7)
2023	38 (26)	1108.0 (0.0/2437.0)	4.3 (0.0/9.9)	53 (49)	1417.0 (198.0/3691.0)	4.3 (0.0/14.4)	43 (10)	349.0 (47.0/598.0)	0.0 (0.0/1.8)

**Table 3:** Estimated number of training years required per resident to fulfill minimum procedural requirements, calculated from national case volume and total trainee numbers. For each category, the number of years required for the employed residents to meet the training requirements was calculated by dividing the reference number of procedures for that category by the annual number of procedures performed per resident. Categories exceeding the timeframe (defined as five years by the training regulations) are highlighted in red.

Procedure Category		2012	2013	2014	2015	2016	2017	2018	2019	2020	2021	2022	2023
101	Eso.-gastro-duodenoscopy	2.1	2.0	2.0	2.2	2.0	2.2	2.0	2.3	2.6	2.2	2.5	2.4
102	Rectosigmoidoscopy	1.6	1.5	1.6	1.8	1.7	2.0	2.0	2.3	2.6	2.6	3.1	3.2
111	Fracture reduction	6.0	6.1	6.7	7.1	6.9	8.1	7.1	8.4	8.7	9.5	7.9	12.0
113	Osteosynth.: diaphyseal	3.1	3.1	2.9	2.8	2.6	2.6	2.4	2.6	2.5	2.7	2.5	2.4
114	Osteosynth.: meta-/epiphyseal	1.6	1.6	1.5	1.5	1.5	1.5	1.4	1.5	1.4	1.4	1.3	1.4
115	Metal implant removal	1.3	1.3	1.4	1.3	1.3	1.3	1.2	1.2	1.2	1.2	1.2	1.5
121	Exc. benign tumors, desm., ear tags	4.7	5.6	5.8	5.7	5.6	6.4	6.4	7.1	8.4	8.0	9.2	8.5
122	Exc. Branch. cysts/fistulas, l.-nodes	5.2	4.8	5.0	5.4	5.3	6.0	5.7	5.9	6.5	7.2	6.1	5.9
131	Thoracotomy as surgical access	3.8	3.7	4.1	4.0	5.4	4.0	4.1	4.5	6.7	4.7	6.3	5.6
132	Complex thor. proc. (1st ass.)	3.3	3.4	3.9	4.1	8.8	4.0	4.4	4.6	9.1	5.4	9.6	7.9
141	Cong. malformation (1st ass.)	6.6	6.1	6.1	6.3	5.9	6.2	6.0	6.7	7.7	7.5	7.6	8.3
142	Abdominal procedures	3.0	3.0	3.0	3.1	3.0	3.1	3.1	3.2	3.3	3.6	3.5	4.1
143	Appendectomy	1.1	1.1	1.2	1.2	1.3	1.3	1.3	1.5	1.4	1.6	1.5	1.8
144	Laparotomy as surgical access	2.6	2.7	2.6	2.6	2.4	2.4	2.5	2.5	2.9	2.7	2.8	3.2
145	Lap. higher complex. (1st ass.)	10.7	12.7	10.5	10.9	12.5	14.0	12.5	14.3	14.3	16.4	17.5	18.7
146	Inguinal herniotomy	2.4	2.4	2.7	2.6	2.7	2.8	2.8	3.1	3.4	3.5	3.6	3.9
147	Abdominal wall hernias	2.7	2.6	2.8	2.9	3.3	3.7	3.8	4.2	5.2	4.8	4.9	4.3
151	Simple urologic procedures	1.6	1.6	1.7	1.7	1.8	1.9	1.8	1.8	2.2	2.0	2.2	2.1
152	Orchidopexy and funicolysis	1.4	1.5	1.5	1.4	1.4	1.5	1.4	1.6	1.7	1.7	1.7	1.8
153	Cystoscopy	1.0	1.0	1.0	1.0	1.1	1.2	1.2	1.2	1.3	1.2	1.2	1.2
154	Complex urol. proc. (1st ass.)	1.9	1.6	1.8	1.7	1.6	1.6	1.2	1.4	1.5	1.5	1.6	1.6
181	Ins. of central ven. access	1.5	1.5	1.5	1.7	1.7	1.9	1.6	1.7	1.5	1.7	2.2	2.0

## Data Availability

The datasets analyzed during this study are publicly available in the repository of the German Federal Joint Committee (Gemeinsamer Bundedausschuss): https://qb-referenzdatenbank.g-ba.de (accessed on 10 February 2026).

## Supplementary Materials

The following supporting information can be downloaded at: https://www.mdpi.com/article/10.3390/children13040554/s1, Table S1: Full list of codes per category used for analysis. Figure S1: Map of pediatric surgery units in Germany by type and units with data.

## Abbreviations

FTE	Full-Time Equivalent
GBA	Gemeinsamer Bundesausschuss (Federal Joint Committee)
MWBO	Musterweiterbildungsordnung (Model Training Regulations)
OPS	Operationen- und Prozedurenschlüssel (Procedure Classification)
