# Supplementary material for: Mind the Gap: A Nationwide Analysis of Case Distribution, Resident Exposure and Institutional Variation in German Pediatric Surgery Training
Source: Children (Basel). 2026 Apr 16;13(4):554. doi: 10.3390/children13040554 (PMC13114497; doi:10.3390/children13040554)
Supplement: Supplementary file 1 [file children-13-00554-s001.zip › children-4231225-supplementary-Heading.pdf]

Supplement Table S1

Full list of codes per category used for analysis

Kat. 101

1-440.9	1-632	5-422.22	5-431.21	5-433.2	5-449.d3
1-440.a	1-632.0	5-422.23	5-431.22	5-433.20	5-449.h3
1-630	1-632.1	5-422.2x	5-431.23	5-433.21	5-449.j3
1-630.0	1-632.x	5-429.j1	5-431.24	5-433.22	5-449.k3
1-630.1	1-632.y	5-429.j4	5-431.25	5-433.23	5-449.m3
1-630.x	1-638.0	5-429.ja	5-431.26	5-433.5	5-449.x3
1-630.y	1-638.1	5-429.jc	5-431.27	5-433.50	5-450.3
1-631	1-638.2	5-429.je	5-431.28	5-433.51	8-100.6
1-631.0	1-638.3	5-429.jg	5-431.29	5-449.43	8-100.7
1-631.1	5-422.2	5-429.k1	5-431.2x	5-449.73	8-100.8
1-631.x	5-422.20	5-431.2	5-431.31	5-449.b3	
1-631.y	5-422.21	5-431.20	5-432.03	5-449.c3	

Kat. 102

1-444	1-652.2	5-452.2x	5-452.71	5-469.n3	5-489.b
1-444.6	1-652.3	5-452.50	5-452.72	5-469.p3	5-489.m
1-444.7	1-652.4	5-452.52	5-452.8	5-482.01	5-492.2
1-444.x	1-653	5-452.5x	5-452.80	5-482.02	5-492.3
1-444.y	1-654	5-452.60	5-452.81	5-482.11	8-100.9
1-557	1-654.0	5-452.61	5-452.82	5-482.12	8-100.a
1-557.0	1-654.1	5-452.62	5-452.b1	5-482.31	
1-557.1	1-654.x	5-452.63	5-469.j3	5-482.32	
1-650.0	1-654.y	5-452.65	5-469.k3	5-482.41	
1-651	5-452.23	5-452.6x	5-469.m3	5-482.42	

Kat. 111

8-200	8-200.4	8-200.c	8-200.h	8-200.s	8-200.x
8-200.0	8-200.5	8-200.d	8-200.j	8-200.t	8-200.y
8-200.1	8-200.6	8-200.e	8-200.k	8-200.u	
8-200.2	8-200.a	8-200.f	8-200.m	8-200.v	
8-200.3	8-200.b	8-200.g	8-200.n	8-200.w	

Kat. 113

5-790.02	5-790.22	5-790.45	5-790.65	5-790.92	5-790.dg
5-790.05	5-790.25	5-790.4g	5-790.68	5-790.95	5-790.dm
5-790.08	5-790.2g	5-790.4m	5-790.6g	5-790.98	5-790.dq
5-790.0g	5-790.2m	5-790.4q	5-790.6m	5-790.9g	5-790.k2
5-790.0m	5-790.2q	5-790.52	5-790.6q	5-790.9m	5-790.k5
5-790.0q	5-790.32	5-790.55	5-790.72	5-790.9q	5-790.k8
5-790.12	5-790.35	5-790.58	5-790.7g	5-790.cg	5-790.kg
5-790.15	5-790.3g	5-790.5g	5-790.7m	5-790.cm	5-790.km
5-790.1g	5-790.3m	5-790.5m	5-790.82	5-790.d2	5-790.kq
5-790.1m	5-790.3q	5-790.5q	5-790.8g	5-790.d5	5-790.m2
5-790.1q	5-790.42	5-790.62	5-790.8m	5-790.d8	5-790.m5

5-790.m8
5-790.mg
5-790.mm
5-790.mq
5-790.n2
5-790.n5
5-790.n8
5-790.ng
5-790.nm
5-790.nq
5-790.p2
5-790.p5
5-790.p8
5-790.pg
5-790.pm
5-790.pq
5-790.x2
5-790.x5
5-790.x8
5-790.xg
5-790.xm
5-790.xq
5-791
5-791.0
5-791.02
5-791.05
5-791.08
5-791.0g
5-791.0m
5-791.0q
5-791.0x
5-791.1
5-791.12
5-791.15
5-791.1g
5-791.1m
5-791.1q
5-791.1x
5-791.2
5-791.22
5-791.25
5-791.28
5-791.2g
5-791.2m
5-791.2q
5-791.2x
5-791.3
5-791.32
5-791.3g
5-791.3m
5-791.3x
5-791.4
5-791.42
5-791.4g
5-791.4x
5-791.5
5-791.52
5-791.55
5-791.5g
5-791.5m
5-791.5q
5-791.5x
5-791.6
5-791.62
5-791.65
5-791.6g
5-791.6m
5-791.6q
5-791.6x
5-791.7
5-791.72
5-791.75
5-791.78
5-791.7g
5-791.7m
5-791.7q
5-791.7x
5-791.8
5-791.82
5-791.85
5-791.88
5-791.8g
5-791.8m
5-791.8q
5-791.8x
5-791.9
5-791.92
5-791.95
5-791.98
5-791.9g
5-791.9m
5-791.9q
5-791.9x
5-791.c
5-791.c2
5-791.c5
5-791.c8
5-791.cg
5-791.cm
5-791.cq
5-791.cx
5-791.d
5-791.d2
5-791.d5
5-791.d8
5-791.dg
5-791.dm
5-791.dq
5-791.dx
5-791.f
5-791.f2
5-791.f5
5-791.f8
5-791.fg
5-791.fm
5-791.fq
5-791.fx
5-791.g
5-791.g2
5-791.g5
5-791.gg
5-791.gm
5-791.gq
5-791.gx
5-791.h
5-791.h2
5-791.h5
5-791.h8
5-791.hg
5-791.hm
5-791.hq
5-791.hx
5-791.k
5-791.k2
5-791.k5
5-791.k8
5-791.kg
5-791.km
5-791.kq
5-791.kx
5-791.m
5-791.m2
5-791.m5
5-791.m8
5-791.mg
5-791.mm
5-791.mq
5-791.mx
5-791.n
5-791.n2
5-791.n5
5-791.n8
5-791.ng
5-791.nm
5-791.nq
5-791.nx
5-791.x
5-791.x2
5-791.x5
5-791.x8
5-791.xg
5-791.xm
5-791.xq
5-791.xx
5-791.y
5-792
5-792.0
5-792.02
5-792.05
5-792.08
5-792.0g
5-792.0m
5-792.0q
5-792.0x
5-792.1
5-792.12
5-792.15
5-792.18
5-792.1g
5-792.1m
5-792.1q
5-792.1x
5-792.2
5-792.22
5-792.25
5-792.28
5-792.2g
5-792.2m
5-792.2q
5-792.2x
5-792.3
5-792.32
5-792.3g
5-792.3m
5-792.3x
5-792.4
5-792.42
5-792.4g
5-792.4x
5-792.5
5-792.52
5-792.55
5-792.58
5-792.5g
5-792.5m
5-792.5q
5-792.5x
5-792.6
5-792.62
5-792.65
5-792.68
5-792.6g
5-792.6m
5-792.6q
5-792.6x
5-792.7
5-792.72
5-792.75
5-792.78
5-792.7g
5-792.7m
5-792.7q
5-792.7x
5-792.8
5-792.82
5-792.85
5-792.88
5-792.8g
5-792.8m
5-792.8q
5-792.8x
5-792.9
5-792.92
5-792.95
5-792.98
5-792.9g
5-792.9m
5-792.9q
5-792.9x
5-792.g
5-792.g2
5-792.g5
5-792.g8
5-792.gg
5-792.gm
5-792.gq
5-792.gx
5-792.h
5-792.h2
5-792.h5
5-792.h8
5-792.hg
5-792.hm
5-792.hq
5-792.hx
5-792.k
5-792.k2
5-792.k5
5-792.k8
5-792.kg
5-792.km
5-792.kq
5-792.kx
5-792.m
5-792.m2
5-792.m5
5-792.m8
5-792.mg
5-792.mm
5-792.mq
5-792.mx
5-792.n
5-792.n2
5-792.n5
5-792.n8
5-792.ng
5-792.nm
5-792.nq
5-792.nx
5-792.x
5-792.x2
5-792.x5
5-792.x8
5-792.xg
5-792.xm
5-792.xq
5-792.xx
5-792.y

Kat. 114

5-790.01	5-790.3r	5-790.81	5-790.kp	5-790.xf	5-793.2f
5-790.03	5-790.41	5-790.8e	5-790.kr	5-790.xh	5-793.2h
5-790.04	5-790.43	5-790.8f	5-790.m1	5-790.xk	5-793.2j
5-790.06	5-790.44	5-790.8h	5-790.m3	5-790.xn	5-793.2k
5-790.07	5-790.46	5-790.8k	5-790.m4	5-790.xp	5-793.2n
5-790.09	5-790.47	5-790.91	5-790.m6	5-790.xr	5-793.2p
5-790.0e	5-790.49	5-790.93	5-790.m7	5-793	5-793.2r
5-790.0f	5-790.4e	5-790.94	5-790.m9	5-793.0	5-793.2x
5-790.0h	5-790.4f	5-790.96	5-790.mf	5-793.01	5-793.3
5-790.0k	5-790.4h	5-790.97	5-790.mh	5-793.03	5-793.31
5-790.0n	5-790.4k	5-790.99	5-790.mk	5-793.04	5-793.33
5-790.0p	5-790.4n	5-790.9e	5-790.mn	5-793.06	5-793.34
5-790.0r	5-790.4p	5-790.9f	5-790.mp	5-793.07	5-793.36
5-790.11	5-790.4r	5-790.9h	5-790.mr	5-793.09	5-793.37
5-790.13	5-790.51	5-790.9k	5-790.n1	5-793.0e	5-793.39
5-790.14	5-790.53	5-790.9n	5-790.n3	5-793.0f	5-793.3e
5-790.16	5-790.54	5-790.9p	5-790.n4	5-793.0h	5-793.3f
5-790.1e	5-790.56	5-790.9r	5-790.n6	5-793.0j	5-793.3h
5-790.1f	5-790.57	5-790.ce	5-790.n7	5-793.0k	5-793.3j
5-790.1h	5-790.59	5-790.cf	5-790.n9	5-793.0n	5-793.3k
5-790.1k	5-790.5e	5-790.ch	5-790.ne	5-793.0p	5-793.3n
5-790.1n	5-790.5f	5-790.ck	5-790.nf	5-793.0r	5-793.3p
5-790.1p	5-790.5h	5-790.cn	5-790.nh	5-793.0x	5-793.3r
5-790.1r	5-790.5k	5-790.d1	5-790.nk	5-793.1	5-793.3x
5-790.21	5-790.5n	5-790.d3	5-790.nn	5-793.11	5-793.4
5-790.23	5-790.5p	5-790.d4	5-790.np	5-793.13	5-793.41
5-790.24	5-790.5r	5-790.d6	5-790.nr	5-793.14	5-793.43
5-790.26	5-790.61	5-790.d7	5-790.p1	5-793.16	5-793.4e
5-790.2e	5-790.63	5-790.d9	5-790.p3	5-793.17	5-793.4f
5-790.2f	5-790.64	5-790.de	5-790.p4	5-793.19	5-793.4h
5-790.2h	5-790.66	5-790.df	5-790.p6	5-793.1e	5-793.4k
5-790.2k	5-790.67	5-790.dh	5-790.p7	5-793.1f	5-793.4n
5-790.2n	5-790.69	5-790.dk	5-790.p9	5-793.1h	5-793.4x
5-790.2p	5-790.6e	5-790.dn	5-790.pe	5-793.1j	5-793.5
5-790.2r	5-790.6f	5-790.dp	5-790.pf	5-793.1k	5-793.51
5-790.31	5-790.6h	5-790.dr	5-790.ph	5-793.1n	5-793.5e
5-790.33	5-790.6k	5-790.k1	5-790.pk	5-793.1p	5-793.5f
5-790.34	5-790.6n	5-790.k3	5-790.pn	5-793.1r	5-793.5h
5-790.36	5-790.6p	5-790.k4	5-790.pp	5-793.1x	5-793.5k
5-790.37	5-790.6r	5-790.k6	5-790.pr	5-793.2	5-793.5x
5-790.39	5-790.71	5-790.k7	5-790.x1	5-793.21	5-793.6
5-790.3e	5-790.73	5-790.k9	5-790.x3	5-793.23	5-793.61
5-790.3f	5-790.7e	5-790.ke	5-790.x4	5-793.24	5-793.63
5-790.3h	5-790.7f	5-790.kf	5-790.x6	5-793.26	5-793.64
5-790.3k	5-790.7h	5-790.kh	5-790.x7	5-793.27	5-793.66
5-790.3n	5-790.7k	5-790.kk	5-790.x9	5-793.29	5-793.67
5-790.3p	5-790.7n	5-790.kn	5-790.xe	5-793.2e	5-793.69

5-793.6e	5-793.9f	5-793.cp	5-793.h7	5-793.n9	5-794.17
5-793.6f	5-793.9h	5-793.cr	5-793.h9	5-793.ne	5-794.19
5-793.6h	5-793.9j	5-793.cx	5-793.he	5-793.nf	5-794.1e
5-793.6j	5-793.9k	5-793.e	5-793.hf	5-793.nh	5-794.1f
5-793.6k	5-793.9n	5-793.ef	5-793.hh	5-793.nj	5-794.1h
5-793.6n	5-793.9p	5-793.eh	5-793.hj	5-793.nk	5-794.1j
5-793.6p	5-793.9r	5-793.ej	5-793.hk	5-793.nn	5-794.1k
5-793.6r	5-793.9x	5-793.ek	5-793.hn	5-793.np	5-794.1n
5-793.6x	5-793.a	5-793.en	5-793.hp	5-793.nr	5-794.1p
5-793.7	5-793.a1	5-793.ep	5-793.hr	5-793.nx	5-794.1r
5-793.71	5-793.a3	5-793.er	5-793.hx	5-793.x	5-794.1x
5-793.73	5-793.a4	5-793.ex	5-793.k	5-793.x1	5-794.2
5-793.74	5-793.a7	5-793.f	5-793.k1	5-793.x3	5-794.21
5-793.76	5-793.a9	5-793.f1	5-793.k3	5-793.x4	5-794.23
5-793.77	5-793.ae	5-793.f3	5-793.k4	5-793.x6	5-794.24
5-793.79	5-793.af	5-793.f4	5-793.k6	5-793.x7	5-794.26
5-793.7e	5-793.ah	5-793.f6	5-793.k7	5-793.x9	5-794.27
5-793.7f	5-793.ak	5-793.f7	5-793.k9	5-793.xe	5-794.29
5-793.7h	5-793.an	5-793.f9	5-793.ke	5-793.xf	5-794.2e
5-793.7j	5-793.ap	5-793.fe	5-793.kf	5-793.xh	5-794.2f
5-793.7k	5-793.ar	5-793.ff	5-793.kh	5-793.xj	5-794.2h
5-793.7n	5-793.ax	5-793.fh	5-793.kj	5-793.xk	5-794.2j
5-793.7p	5-793.b	5-793.fj	5-793.kk	5-793.xn	5-794.2k
5-793.7r	5-793.b1	5-793.fk	5-793.kn	5-793.xp	5-794.2n
5-793.7x	5-793.b3	5-793.fn	5-793.kp	5-793.xr	5-794.2p
5-793.8	5-793.b4	5-793.fp	5-793.kr	5-793.xx	5-794.2r
5-793.81	5-793.b6	5-793.fr	5-793.kx	5-793.y	5-794.2x
5-793.83	5-793.b7	5-793.fx	5-793.m	5-794	5-794.3
5-793.84	5-793.b9	5-793.g	5-793.m1	5-794.0	5-794.31
5-793.86	5-793.be	5-793.g1	5-793.m3	5-794.01	5-794.33
5-793.87	5-793.bf	5-793.g3	5-793.m4	5-794.03	5-794.3e
5-793.89	5-793.bh	5-793.g4	5-793.m6	5-794.04	5-794.3f
5-793.8e	5-793.bk	5-793.g6	5-793.m7	5-794.06	5-794.3h
5-793.8f	5-793.bn	5-793.g7	5-793.m9	5-794.07	5-794.3k
5-793.8h	5-793.bp	5-793.g9	5-793.me	5-794.09	5-794.3n
5-793.8j	5-793.br	5-793.ge	5-793.mf	5-794.0e	5-794.3x
5-793.8k	5-793.bx	5-793.gf	5-793.mh	5-794.0f	5-794.4
5-793.8n	5-793.c	5-793.gh	5-793.mj	5-794.0h	5-794.41
5-793.8p	5-793.c1	5-793.gj	5-793.mk	5-794.0j	5-794.43
5-793.8r	5-793.c3	5-793.gk	5-793.mn	5-794.0k	5-794.4e
5-793.8x	5-793.c4	5-793.gn	5-793.mp	5-794.0n	5-794.4f
5-793.9	5-793.c6	5-793.gp	5-793.mr	5-794.0p	5-794.4h
5-793.91	5-793.c7	5-793.gr	5-793.mx	5-794.0r	5-794.4k
5-793.93	5-793.c9	5-793.gx	5-793.n	5-794.0x	5-794.4x
5-793.94	5-793.ce	5-793.h	5-793.n1	5-794.1	5-794.5
5-793.96	5-793.cf	5-793.h1	5-793.n3	5-794.11	5-794.51
5-793.97	5-793.ch	5-793.h3	5-793.n4	5-794.13	5-794.53
5-793.99	5-793.ck	5-793.h4	5-793.n6	5-794.14	5-794.54
5-793.9e	5-793.cn	5-793.h6	5-793.n7	5-794.16	5-794.56

5-794.57	5-794.7n	5-794.b6	5-794.f6	5-794.hk	5-794.n3
5-794.59	5-794.7p	5-794.b7	5-794.f7	5-794.hn	5-794.n4
5-794.5e	5-794.7r	5-794.b9	5-794.f9	5-794.hp	5-794.n6
5-794.5f	5-794.7x	5-794.be	5-794.fe	5-794.hr	5-794.n7
5-794.5h	5-794.8	5-794.bf	5-794.ff	5-794.hx	5-794.n9
5-794.5j	5-794.81	5-794.bh	5-794.fh	5-794.k	5-794.ne
5-794.5k	5-794.83	5-794.bk	5-794.fj	5-794.k1	5-794.nf
5-794.5n	5-794.84	5-794.bn	5-794.fk	5-794.k3	5-794.nh
5-794.5p	5-794.86	5-794.bp	5-794.fn	5-794.k4	5-794.nj
5-794.5r	5-794.87	5-794.br	5-794.fp	5-794.k6	5-794.nk
5-794.5x	5-794.89	5-794.bx	5-794.fr	5-794.k7	5-794.nn
5-794.6	5-794.8e	5-794.c	5-794.fx	5-794.k9	5-794.np
5-794.61	5-794.8f	5-794.c1	5-794.g	5-794.ke	5-794.nr
5-794.63	5-794.8h	5-794.c3	5-794.g1	5-794.kf	5-794.nx
5-794.64	5-794.8j	5-794.c4	5-794.g3	5-794.kh	5-794.x
5-794.66	5-794.8k	5-794.c6	5-794.g4	5-794.kj	5-794.x1
5-794.67	5-794.8n	5-794.c7	5-794.g6	5-794.kk	5-794.x3
5-794.69	5-794.8p	5-794.c9	5-794.g7	5-794.kn	5-794.x4
5-794.6e	5-794.8r	5-794.ce	5-794.g9	5-794.kp	5-794.x6
5-794.6f	5-794.8x	5-794.cf	5-794.ge	5-794.kr	5-794.x7
5-794.6h	5-794.a	5-794.ch	5-794.gf	5-794.kx	5-794.x9
5-794.6j	5-794.a1	5-794.ck	5-794.gh	5-794.m	5-794.xe
5-794.6k	5-794.a3	5-794.cn	5-794.gj	5-794.m1	5-794.xf
5-794.6n	5-794.a4	5-794.cp	5-794.gk	5-794.m3	5-794.xh
5-794.6p	5-794.a6	5-794.cr	5-794.gn	5-794.m4	5-794.xj
5-794.6r	5-794.a7	5-794.cx	5-794.gp	5-794.m6	5-794.xk
5-794.6x	5-794.a9	5-794.e	5-794.gr	5-794.m7	5-794.xn
5-794.7	5-794.ae	5-794.ef	5-794.gx	5-794.m9	5-794.xp
5-794.71	5-794.af	5-794.eh	5-794.h	5-794.me	5-794.xr
5-794.73	5-794.ah	5-794.ej	5-794.h1	5-794.mf	5-794.xx
5-794.74	5-794.ak	5-794.ek	5-794.h3	5-794.mh	5-794.y
5-794.76	5-794.an	5-794.en	5-794.h4	5-794.mj	5-799.1
5-794.77	5-794.ap	5-794.ep	5-794.h6	5-794.mk	5-799.2
5-794.79	5-794.ar	5-794.er	5-794.h7	5-794.mn	5-799.3
5-794.7e	5-794.ax	5-794.ex	5-794.h9	5-794.mp	5-799.4
5-794.7f	5-794.b	5-794.f	5-794.he	5-794.mr	5-799.x
5-794.7h	5-794.b1	5-794.f1	5-794.hf	5-794.mx	
5-794.7j	5-794.b3	5-794.f3	5-794.hh	5-794.n	
5-794.7k	5-794.b4	5-794.f4	5-794.hj	5-794.n1	

Kat 115

5-349.3	5-787.05	5-787.0g	5-787.0r	5-787.1	5-787.17
5-787	5-787.06	5-787.0h	5-787.0s	5-787.10	5-787.18
5-787.0	5-787.0a	5-787.0j	5-787.0t	5-787.11	5-787.19
5-787.00	5-787.0b	5-787.0k	5-787.0u	5-787.12	5-787.1a
5-787.01	5-787.0c	5-787.0m	5-787.0v	5-787.13	5-787.1b
5-787.02	5-787.0d	5-787.0n	5-787.0w	5-787.14	5-787.1c
5-787.03	5-787.0e	5-787.0p	5-787.0x	5-787.15	5-787.1d
5-787.04	5-787.0f	5-787.0q	5-787.0z	5-787.16	5-787.1e

5-787.1f	5-787.2z	5-787.5	5-787.6r	5-787.98	5-787.cx
5-787.1g	5-787.3	5-787.50	5-787.6x	5-787.99	5-787.e
5-787.1h	5-787.30	5-787.51	5-787.7	5-787.9a	5-787.e0
5-787.1j	5-787.31	5-787.52	5-787.71	5-787.9b	5-787.e1
5-787.1k	5-787.32	5-787.53	5-787.72	5-787.9c	5-787.e2
5-787.1m	5-787.33	5-787.54	5-787.73	5-787.9d	5-787.e3
5-787.1n	5-787.34	5-787.55	5-787.74	5-787.9e	5-787.e4
5-787.1p	5-787.35	5-787.56	5-787.75	5-787.9f	5-787.e5
5-787.1q	5-787.36	5-787.57	5-787.7e	5-787.9g	5-787.e6
5-787.1r	5-787.37	5-787.58	5-787.7f	5-787.9h	5-787.e7
5-787.1s	5-787.38	5-787.59	5-787.7g	5-787.9j	5-787.e8
5-787.1t	5-787.39	5-787.5a	5-787.7h	5-787.9k	5-787.e9
5-787.1u	5-787.3a	5-787.5b	5-787.7k	5-787.9m	5-787.ea
5-787.1v	5-787.3b	5-787.5c	5-787.7m	5-787.9n	5-787.eb
5-787.1w	5-787.3c	5-787.5d	5-787.7n	5-787.9p	5-787.ec
5-787.1x	5-787.3d	5-787.5e	5-787.7p	5-787.9q	5-787.ed
5-787.1z	5-787.3e	5-787.5f	5-787.7q	5-787.9r	5-787.ee
5-787.2	5-787.3f	5-787.5g	5-787.7r	5-787.9s	5-787.ef
5-787.20	5-787.3g	5-787.5h	5-787.7x	5-787.9t	5-787.eg
5-787.21	5-787.3h	5-787.5j	5-787.8	5-787.9u	5-787.eh
5-787.22	5-787.3j	5-787.5k	5-787.81	5-787.9v	5-787.ej
5-787.23	5-787.3k	5-787.5m	5-787.82	5-787.9w	5-787.ek
5-787.24	5-787.3m	5-787.5n	5-787.83	5-787.9x	5-787.em
5-787.25	5-787.3n	5-787.5p	5-787.84	5-787.9z	5-787.en
5-787.26	5-787.3p	5-787.5q	5-787.85	5-787.c	5-787.ep
5-787.27	5-787.3q	5-787.5r	5-787.86	5-787.c1	5-787.eq
5-787.28	5-787.3r	5-787.5s	5-787.8b	5-787.c2	5-787.er
5-787.29	5-787.3s	5-787.5t	5-787.8e	5-787.c3	5-787.es
5-787.2a	5-787.3t	5-787.5u	5-787.8f	5-787.c4	5-787.et
5-787.2b	5-787.3u	5-787.5v	5-787.8g	5-787.c5	5-787.eu
5-787.2c	5-787.3v	5-787.5w	5-787.8h	5-787.c6	5-787.ev
5-787.2d	5-787.3w	5-787.5x	5-787.8k	5-787.c7	5-787.ew
5-787.2e	5-787.3x	5-787.5z	5-787.8m	5-787.c8	5-787.ex
5-787.2f	5-787.3z	5-787.6	5-787.8n	5-787.c9	5-787.ez
5-787.2g	5-787.4	5-787.61	5-787.8p	5-787.ca	5-787.g
5-787.2h	5-787.40	5-787.62	5-787.8q	5-787.cb	5-787.g0
5-787.2j	5-787.41	5-787.63	5-787.8r	5-787.cd	5-787.g1
5-787.2k	5-787.42	5-787.64	5-787.8t	5-787.ce	5-787.g2
5-787.2m	5-787.43	5-787.65	5-787.8v	5-787.cf	5-787.g3
5-787.2n	5-787.4d	5-787.66	5-787.8x	5-787.cg	5-787.g4
5-787.2p	5-787.4e	5-787.6e	5-787.9	5-787.ch	5-787.g5
5-787.2q	5-787.4f	5-787.6f	5-787.90	5-787.ck	5-787.g6
5-787.2r	5-787.4g	5-787.6g	5-787.91	5-787.cm	5-787.ga
5-787.2s	5-787.4h	5-787.6h	5-787.92	5-787.cn	5-787.gb
5-787.2t	5-787.4k	5-787.6k	5-787.93	5-787.cp	5-787.gc
5-787.2u	5-787.4m	5-787.6m	5-787.94	5-787.cq	5-787.ge
5-787.2v	5-787.4n	5-787.6n	5-787.95	5-787.cr	5-787.gf
5-787.2w	5-787.4x	5-787.6p	5-787.96	5-787.cs	5-787.gg
5-787.2x	5-787.4z	5-787.6q	5-787.97	5-787.ct	5-787.gh

5-787.gj	5-787.k8	5-787.m2	5-787.n0	5-787.nt	5-787.xd
5-787.gk	5-787.k9	5-787.m3	5-787.n1	5-787.nu	5-787.xe
5-787.gm	5-787.ka	5-787.m4	5-787.n2	5-787.nv	5-787.xf
5-787.gn	5-787.kb	5-787.m5	5-787.n3	5-787.nw	5-787.xg
5-787.gp	5-787.kc	5-787.m6	5-787.n4	5-787.nx	5-787.xh
5-787.gq	5-787.kd	5-787.m7	5-787.n5	5-787.nz	5-787.xj
5-787.gr	5-787.ke	5-787.m8	5-787.n6	5-787.p	5-787.xk
5-787.gs	5-787.kf	5-787.m9	5-787.n7	5-787.ps	5-787.xm
5-787.gt	5-787.kg	5-787.ma	5-787.n8	5-787.pt	5-787.xn
5-787.gu	5-787.kh	5-787.mb	5-787.n9	5-787.pu	5-787.xp
5-787.gv	5-787.kj	5-787.md	5-787.na	5-787.pv	5-787.xq
5-787.gw	5-787.kk	5-787.me	5-787.nb	5-787.px	5-787.xr
5-787.gx	5-787.km	5-787.mf	5-787.nc	5-787.x	5-787.xs
5-787.gz	5-787.kn	5-787.mg	5-787.nd	5-787.x0	5-787.xt
5-787.j	5-787.kp	5-787.mh	5-787.ne	5-787.x1	5-787.xu
5-787.j0	5-787.kq	5-787.mk	5-787.nf	5-787.x2	5-787.xv
5-787.j1	5-787.kr	5-787.mm	5-787.ng	5-787.x3	5-787.xw
5-787.k	5-787.ks	5-787.mn	5-787.nh	5-787.x4	5-787.xx
5-787.k0	5-787.kt	5-787.mp	5-787.nj	5-787.x5	5-787.xz
5-787.k1	5-787.ku	5-787.mq	5-787.nk	5-787.x6	5-787.y
5-787.k2	5-787.kv	5-787.mr	5-787.nm	5-787.x7	
5-787.k3	5-787.kw	5-787.ms	5-787.nn	5-787.x8	
5-787.k4	5-787.kx	5-787.mt	5-787.np	5-787.x9	
5-787.k5	5-787.kz	5-787.mu	5-787.nq	5-787.xa	
5-787.k6	5-787.m	5-787.mv	5-787.nr	5-787.xb	
5-787.k7	5-787.m1	5-787.n	5-787.ns	5-787.xc	

Kat. 121

5-181	5-181.4	5-182.0	5-261.2	5-273.7	5-894.x4
5-181.0	5-181.5	5-182.x	5-261.5	5-273.8	5-895.04
5-181.1	5-181.6	5-182.y	5-273.5	5-894.04	5-895.24
5-181.3	5-181.7	5-212.0	5-273.6	5-894.14	5-895.x4

Kat 122

1-586.0	5-065.5	5-291.3	5-401.03	5-403.04	5-403.30
5-065	5-065.x	5-291.x	5-401.0x	5-403.05	5-403.31
5-065.0	5-065.y	5-291.y	5-402.0	5-403.10	5-403.32
5-065.1	5-291	5-401.0	5-403.00	5-403.11	5-403.x
5-065.2	5-291.0	5-401.00	5-403.01	5-403.12	
5-065.3	5-291.1	5-401.01	5-403.02	5-403.20	
5-065.4	5-291.2	5-401.02	5-403.03	5-403.22	

Kat. 131

1-559.0	1-581.4	1-691.0	5-322.c7	5-322.e4	5-322.g
1-581	1-581.x	1-691.1	5-322.c8	5-322.e5	5-322.g1
1-581.0	1-581.y	5-068.0	5-322.ca	5-322.f	5-322.g2
1-581.1	1-586.3	5-322.c	5-322.d	5-322.f4	5-322.x
1-581.2	1-587.2	5-322.c4	5-322.d1	5-322.f5	5-322.y
1-581.3	1-691	5-322.c5	5-322.d2	5-322.f7	5-333.0

5-333.1	5-340.2	5-340.9	5-340.y	5-401.2x	5-401.e
5-333.x	5-340.5	5-340.c	5-344.10	5-401.70	
5-333.y	5-340.7	5-340.d	5-349.5	5-401.72	
5-340.1	5-340.8	5-340.x	5-401.20	5-401.d	

Kat 132

5-043.1	5-316.61	5-322.ec	5-323.7	5-324.72	5-325.0x
5-077	5-316.6x	5-322.ed	5-323.71	5-324.73	5-325.1
5-077.0	5-316.7	5-322.ee	5-323.72	5-324.74	5-325.11
5-077.1	5-316.8	5-322.ef	5-323.73	5-324.7x	5-325.12
5-077.2	5-316.x	5-322.f6	5-323.8	5-324.8	5-325.13
5-077.3	5-316.y	5-322.f8	5-323.81	5-324.81	5-325.14
5-077.4	5-320.1	5-322.f9	5-323.82	5-324.8x	5-325.15
5-077.5	5-320.2	5-322.fa	5-323.83	5-324.9	5-325.16
5-077.x	5-321	5-322.fb	5-323.9	5-324.91	5-325.17
5-077.y	5-321.0	5-322.fc	5-323.91	5-324.9x	5-325.18
5-078	5-321.1	5-322.fd	5-323.92	5-324.a	5-325.1x
5-078.0	5-321.2	5-322.fe	5-323.93	5-324.a1	5-325.2
5-078.x	5-321.3	5-322.ff	5-323.a	5-324.a2	5-325.21
5-078.y	5-321.4	5-322.fg	5-323.a1	5-324.a3	5-325.22
5-314.0	5-321.40	5-322.fh	5-323.a2	5-324.a4	5-325.23
5-314.00	5-321.41	5-322.fj	5-323.a3	5-324.a5	5-325.24
5-314.01	5-321.42	5-322.g3	5-323.b	5-324.ax	5-325.25
5-314.02	5-321.43	5-322.h	5-323.b1	5-324.b	5-325.26
5-314.0x	5-321.44	5-322.h4	5-323.b2	5-324.b1	5-325.27
5-314.1	5-321.45	5-322.h5	5-323.b3	5-324.b2	5-325.28
5-314.11	5-321.46	5-322.h6	5-323.x	5-324.b3	5-325.2x
5-314.12	5-321.4x	5-322.h7	5-323.x1	5-324.b4	5-325.3
5-314.13	5-321.x	5-322.h8	5-323.x2	5-324.b5	5-325.31
5-314.1x	5-321.y	5-322.h9	5-323.x3	5-324.bx	5-325.32
5-314.2	5-322	5-322.ha	5-323.y	5-324.c	5-325.33
5-314.20	5-322.c6	5-322.hb	5-324	5-324.x	5-325.34
5-314.21	5-322.c9	5-322.hc	5-324.2	5-324.x1	5-325.35
5-314.22	5-322.cb	5-322.hd	5-324.21	5-324.x2	5-325.36
5-314.2x	5-322.cc	5-322.he	5-324.22	5-324.x3	5-325.37
5-314.31	5-322.cd	5-322.hf	5-324.23	5-324.x4	5-325.38
5-314.x	5-322.ce	5-323	5-324.2x	5-324.x5	5-325.3x
5-314.y	5-322.cf	5-323.4	5-324.3	5-324.xx	5-325.4
5-316	5-322.cg	5-323.41	5-324.31	5-324.y	5-325.41
5-316.0	5-322.ch	5-323.42	5-324.32	5-325	5-325.42
5-316.1	5-322.cj	5-323.43	5-324.33	5-325.0	5-325.43
5-316.4	5-322.d3	5-323.5	5-324.34	5-325.01	5-325.44
5-316.5	5-322.e	5-323.51	5-324.3x	5-325.02	5-325.4x
5-316.50	5-322.e6	5-323.52	5-324.6	5-325.03	5-325.5
5-316.51	5-322.e7	5-323.53	5-324.61	5-325.04	5-325.51
5-316.52	5-322.e8	5-323.6	5-324.62	5-325.05	5-325.52
5-316.5x	5-322.e9	5-323.61	5-324.6x	5-325.06	5-325.53
5-316.6	5-322.ea	5-323.62	5-324.7	5-325.07	5-325.54
5-316.60	5-322.eb	5-323.63	5-324.71	5-325.08	5-325.55

5-325.56	5-325.x5	5-328.36	5-334.8	5-343.6	5-346.a3
5-325.57	5-325.x6	5-328.37	5-334.x	5-343.7	5-346.a4
5-325.58	5-325.x7	5-328.38	5-334.y	5-344	5-346.a5
5-325.5x	5-325.x8	5-328.3x	5-339.1	5-344.0	5-346.a6
5-325.6	5-325.xx	5-328.4	5-339.2	5-344.1	5-346.ax
5-325.61	5-325.y	5-328.41	5-339.61	5-344.11	5-346.b
5-325.62	5-327	5-328.42	5-339.65	5-344.12	5-346.c
5-325.63	5-327.0	5-328.43	5-340.a	5-344.13	5-346.c0
5-325.64	5-327.1	5-328.44	5-340.b	5-344.2	5-346.c1
5-325.65	5-327.2	5-328.45	5-341	5-344.3	5-346.c2
5-325.66	5-327.3	5-328.46	5-341.1	5-344.4	5-346.c3
5-325.67	5-327.4	5-328.47	5-341.2	5-344.40	5-346.cx
5-325.68	5-327.5	5-328.48	5-341.20	5-344.41	5-346.d
5-325.6x	5-327.7	5-328.4x	5-341.21	5-344.42	5-346.d0
5-325.7	5-327.8	5-328.5	5-341.22	5-344.43	5-346.d1
5-325.71	5-327.x	5-328.51	5-341.23	5-344.5	5-346.d2
5-325.72	5-327.y	5-328.52	5-341.2x	5-344.x	5-346.d3
5-325.73	5-328	5-328.53	5-341.3	5-344.y	5-346.dx
5-325.74	5-328.0	5-328.5x	5-341.30	5-345	5-346.x
5-325.75	5-328.01	5-328.6	5-341.31	5-345.0	5-346.y
5-325.76	5-328.02	5-328.x	5-341.32	5-345.1	5-347.2
5-325.77	5-328.03	5-328.x1	5-341.33	5-345.2	5-357.0
5-325.78	5-328.04	5-328.x2	5-341.3x	5-345.3	5-399.0
5-325.7x	5-328.05	5-328.x3	5-341.x	5-345.4	5-402.c
5-325.8	5-328.06	5-328.x4	5-341.y	5-345.5	5-402.d
5-325.81	5-328.07	5-328.x5	5-342	5-346.1	5-402.e
5-325.82	5-328.08	5-328.x6	5-342.0	5-346.2	5-402.f
5-325.83	5-328.0x	5-328.x7	5-342.01	5-346.3	5-404.1
5-325.84	5-328.1	5-328.x8	5-342.02	5-346.4	5-404.8
5-325.85	5-328.11	5-328.xx	5-342.03	5-346.5	5-404.j
5-325.86	5-328.12	5-328.y	5-342.0x	5-346.6	5-404.k
5-325.87	5-328.13	5-329	5-342.1	5-346.60	5-405.0
5-325.88	5-328.14	5-330	5-342.11	5-346.61	5-405.1
5-325.8x	5-328.15	5-330.0	5-342.12	5-346.62	5-405.2
5-325.9	5-328.16	5-330.1	5-342.13	5-346.63	5-405.x
5-325.91	5-328.17	5-330.2	5-342.14	5-346.6x	5-420.01
5-325.92	5-328.18	5-330.x	5-342.15	5-346.7	5-420.03
5-325.93	5-328.1x	5-330.y	5-342.16	5-346.8	5-420.05
5-325.94	5-328.2	5-331	5-342.17	5-346.80	5-420.11
5-325.95	5-328.21	5-333	5-342.18	5-346.81	5-420.21
5-325.96	5-328.22	5-334	5-342.19	5-346.82	5-420.23
5-325.97	5-328.23	5-334.0	5-342.1x	5-346.8x	5-420.25
5-325.98	5-328.2x	5-334.1	5-342.2	5-346.9	5-420.x1
5-325.9x	5-328.3	5-334.2	5-342.x	5-346.90	5-420.x3
5-325.x	5-328.31	5-334.3	5-342.y	5-346.91	5-420.x5
5-325.x1	5-328.32	5-334.4	5-343.2	5-346.a	5-421
5-325.x2	5-328.33	5-334.5	5-343.3	5-346.a0	5-421.x
5-325.x3	5-328.34	5-334.6	5-343.4	5-346.a1	5-421.y
5-325.x4	5-328.35	5-334.7	5-343.5	5-346.a2	5-422.0

5-422.1
5-423.1
5-423.2
5-424.0
5-424.1
5-424.10
5-424.11
5-424.12
5-424.1x
5-424.x
5-424.y
5-425.x
5-425.y
5-426.01
5-426.0x
5-426.1
5-426.11
5-426.1x
5-426.2
5-426.21
5-426.x1
5-426.xx
5-426.y
5-427.0
5-427.1
5-429.0
5-429.n
5-448.x1

Kat 141

5-427
5-427.01
5-427.02
5-427.03
5-427.0x
5-427.11
5-427.12
5-427.13
5-427.14
5-427.1x
5-427.2
5-427.21
5-427.22
5-427.23
5-427.24
5-427.2x
5-427.x
5-427.x1
5-427.x2
5-427.x3
5-427.x4
5-427.xx
5-427.y
5-428
5-428.0
5-428.1
5-428.2
5-428.3
5-428.4
5-428.5
5-428.6
5-428.7
5-428.x
5-428.y
5-449
5-449.01
5-449.22
5-449.3
5-449.30
5-449.31
5-449.32
5-449.3x
5-451.0
5-456.01
5-456.02
5-456.03
5-456.04
5-456.05
5-456.06
5-456.07
5-456.08
5-456.0x
5-456.10
5-456.11
5-456.12
5-456.13
5-456.14
5-456.15
5-456.17
5-456.18
5-456.1x
5-456.20
5-456.22
5-456.23
5-456.24
5-456.25
5-456.26
5-456.28
5-456.x0
5-456.x1
5-456.x2
5-456.x3
5-456.x4
5-456.x5
5-456.x6
5-456.x7
5-456.xx
5-469.50
5-469.51
5-469.52
5-469.5x
5-469.6
5-469.60
5-469.61
5-469.62
5-469.6x
5-482.9x
5-482.b0
5-484
5-484.01
5-484.02
5-484.05
5-484.06
5-484.08
5-484.09
5-484.0x
5-484.1
5-484.11
5-484.12
5-484.15
5-484.16
5-484.18
5-484.19
5-484.1x
5-484.2
5-484.21
5-484.22
5-484.25
5-484.26
5-484.27
5-484.28
5-484.29
5-484.2x
5-484.3
5-484.31
5-484.32
5-484.35
5-484.36
5-484.38
5-484.39
5-484.3x
5-484.4
5-484.41
5-484.42
5-484.45
5-484.46
5-484.4x
5-484.5
5-484.51
5-484.52
5-484.55
5-484.56
5-484.58
5-484.59
5-484.5x
5-484.6
5-484.61
5-484.65
5-484.68
5-484.6x
5-484.7
5-484.71
5-484.72
5-484.75
5-484.76
5-484.7x
5-484.x
5-484.x1
5-484.x2
5-484.x5
5-484.x6
5-484.x8
5-484.x9
5-484.xx
5-484.y
5-495
5-495.0
5-495.00
5-495.01
5-495.02
5-495.0x
5-495.1
5-495.10
5-495.11
5-495.12
5-495.1x
5-495.2
5-495.20
5-495.21
5-495.22
5-495.2x
5-495.3
5-495.30
5-495.31
5-495.32
5-495.3x
5-495.4
5-495.40
5-495.42
5-495.4x
5-495.5
5-495.6
5-495.x
5-495.x0
5-495.x1
5-495.x2
5-495.xx
5-495.y
5-496.3
5-496.5
5-496.60
5-496.61
5-496.62
5-496.6x
5-496.b
5-537
5-537.0
5-537.1
5-537.2
5-537.3
5-537.4
5-537.5
5-537.x
5-537.y
5-538
5-538.0
5-538.1
5-538.2
5-538.3
5-538.4
5-538.40
5-538.41
5-538.43
5-538.44
5-538.45
5-538.4x
5-538.5
5-538.6
5-538.7
5-538.8
5-538.9
5-538.90
5-538.91
5-538.93
5-538.94
5-538.95
5-538.9x
5-538.a
5-538.b
5-538.x
5-538.y

Kat 142

1-550	5-432.00	5-449.e1	5-454.8	5-460.1	5-461.51
1-551.0	5-432.01	5-449.x1	5-454.x	5-460.10	5-461.52
1-551.1	5-432.02	5-449.x2	5-454.y	5-460.11	5-461.6
1-551.x	5-432.0x	5-451.1	5-455	5-460.12	5-461.60
1-551.y	5-432.1	5-451.2	5-455.0	5-460.2	5-461.61
1-552.0	5-432.x	5-451.50	5-455.01	5-460.20	5-461.62
1-552.1	5-432.y	5-451.5x	5-455.02	5-460.21	5-461.7
1-553	5-433.3x	5-451.b0	5-455.03	5-460.22	5-461.70
1-554	5-433.40	5-451.b1	5-455.04	5-460.3	5-461.71
1-555	5-433.4x	5-452.0	5-455.05	5-460.30	5-461.72
1-555.0	5-433.60	5-452.1	5-455.06	5-460.31	5-461.x
1-555.1	5-433.x	5-452.40	5-455.07	5-460.32	5-461.x0
1-555.2	5-448.00	5-452.42	5-455.0x	5-460.4	5-461.x1
1-556	5-448.01	5-452.4x	5-455.1	5-460.40	5-461.x2
1-556.0	5-448.02	5-452.a0	5-455.11	5-460.41	5-461.y
1-556.1	5-448.03	5-453.0	5-455.12	5-460.42	5-463
1-556.2	5-448.0x	5-453.1	5-455.13	5-460.5	5-463.0
1-556.3	5-448.1	5-453.2	5-455.14	5-460.50	5-463.00
1-559	5-448.10	5-454	5-455.15	5-460.51	5-463.01
1-559.1	5-448.11	5-454.0	5-455.16	5-460.52	5-463.02
1-559.2	5-448.12	5-454.00	5-455.17	5-460.x	5-463.1
1-559.3	5-448.13	5-454.01	5-455.1x	5-460.x0	5-463.10
1-559.4	5-448.1x	5-454.02	5-455.2	5-460.x1	5-463.11
1-694	5-448.22	5-454.1	5-455.21	5-460.x2	5-463.12
5-347.7	5-448.23	5-454.10	5-455.22	5-460.y	5-463.2
5-401.80	5-448.30	5-454.11	5-455.23	5-461	5-463.20
5-401.8x	5-448.32	5-454.12	5-455.24	5-461.0	5-463.21
5-401.90	5-448.33	5-454.2	5-455.25	5-461.00	5-463.22
5-401.9x	5-448.3x	5-454.20	5-455.26	5-461.01	5-463.3
5-401.a0	5-448.4	5-454.21	5-455.27	5-461.02	5-463.30
5-401.j	5-448.40	5-454.22	5-455.2x	5-461.1	5-463.31
5-408.21	5-448.41	5-454.3	5-455.3	5-461.10	5-463.32
5-408.4	5-448.42	5-454.30	5-455.31	5-461.11	5-463.x
5-408.81	5-448.43	5-454.31	5-455.35	5-461.12	5-463.x0
5-430.0	5-448.4x	5-454.32	5-455.37	5-461.2	5-463.x1
5-430.1	5-448.5	5-454.4	5-459	5-461.20	5-463.x2
5-430.x	5-448.50	5-454.40	5-459.0	5-461.21	5-463.y
5-430.y	5-448.51	5-454.41	5-459.1	5-461.22	5-464
5-431	5-448.52	5-454.42	5-459.2	5-461.3	5-464.0
5-431.0	5-448.53	5-454.5	5-459.3	5-461.30	5-464.00
5-431.1	5-448.5x	5-454.50	5-459.4	5-461.31	5-464.01
5-431.3	5-448.6	5-454.51	5-459.x	5-461.32	5-464.02
5-431.30	5-448.60	5-454.52	5-459.y	5-461.4	5-464.03
5-431.3x	5-448.61	5-454.6	5-460	5-461.40	5-464.0x
5-431.x	5-448.62	5-454.60	5-460.0	5-461.41	5-464.1
5-431.y	5-448.63	5-454.61	5-460.00	5-461.42	5-464.10
5-432	5-448.6x	5-454.62	5-460.01	5-461.5	5-464.11
5-432.0	5-448.x2	5-454.7	5-460.02	5-461.50	5-464.12

5-464.13	5-465.y	5-468.10	5-510.0	5-514.dx	5-651.a1
5-464.1x	5-466	5-468.11	5-510.1	5-514.x1	5-651.a2
5-464.2	5-466.0	5-468.12	5-510.2	5-543.0	5-651.b1
5-464.20	5-466.1	5-468.13	5-510.3	5-543.1	5-651.b2
5-464.21	5-466.2	5-468.1x	5-510.4	5-543.2	5-651.x1
5-464.22	5-466.x	5-469.0x	5-510.40	5-543.20	5-651.x2
5-464.23	5-466.y	5-469.10	5-510.41	5-543.21	5-652.41
5-464.2x	5-467.10	5-469.11	5-510.42	5-543.3	5-652.42
5-464.31	5-467.11	5-469.12	5-510.4x	5-543.40	5-652.51
5-464.32	5-467.12	5-469.1x	5-511	5-543.41	5-652.52
5-464.33	5-467.13	5-469.30	5-511.0	5-543.42	5-652.61
5-464.3x	5-467.1x	5-469.31	5-511.01	5-543.x	5-652.62
5-464.4	5-467.30	5-469.32	5-511.02	5-543.y	5-653.22
5-464.40	5-467.31	5-469.3x	5-511.1	5-546.1	5-653.31
5-464.41	5-467.32	5-469.40	5-511.11	5-549.0	5-653.32
5-464.42	5-467.33	5-469.7	5-511.12	5-549.2	5-656.81
5-464.43	5-467.3x	5-469.70	5-511.2	5-549.20	5-656.82
5-464.4x	5-467.40	5-469.71	5-511.21	5-549.21	5-656.91
5-464.5	5-467.41	5-469.72	5-511.22	5-549.2x	5-656.92
5-464.50	5-467.42	5-469.7x	5-511.3	5-549.3	5-656.a1
5-464.51	5-467.43	5-469.b1	5-511.4	5-549.30	5-656.a2
5-464.52	5-467.4x	5-469.b2	5-511.41	5-549.5	5-656.b1
5-464.53	5-467.50	5-469.h0	5-511.42	5-549.80	5-656.b2
5-464.5x	5-467.51	5-469.h1	5-511.5	5-549.81	5-656.x1
5-464.x0	5-467.52	5-469.hx	5-511.51	5-549.8x	5-656.x2
5-464.x1	5-467.53	5-469.j0	5-511.52	5-549.d0	5-659.22
5-464.x2	5-467.5x	5-469.k1	5-511.x	5-549.d1	5-659.x2
5-464.x3	5-468	5-469.n0	5-511.y	5-549.dx	5-660.4
5-464.xx	5-468.0	5-469.n1	5-514.01	5-622.4	5-661.42
5-464.y	5-468.00	5-469.px	5-514.02	5-650.3	5-661.52
5-465	5-468.01	5-469.x1	5-514.21	5-650.4	5-661.62
5-465.0	5-468.02	5-469.x2	5-514.31	5-651.81	5-665.42
5-465.1	5-468.03	5-500.0	5-514.51	5-651.82	5-665.52
5-465.2	5-468.0x	5-500.1	5-514.b1	5-651.91	5-665.x2
5-465.x	5-468.1	5-510	5-514.d0	5-651.92	

Kat 143

5-470	5-470.11	5-470.y	5-471.10	5-471.y	5-479.x
5-470.0	5-470.1x	5-471	5-471.11	5-479	5-479.y
5-470.1	5-470.2	5-471.0	5-471.1x	5-479.0	
5-470.10	5-470.x	5-471.1	5-471.x	5-479.1	

Kat 144

	1-570.2	1-586.7	5-043.2	5-401.40	5-448.20
1-570	1-571.0	1-586.8	5-401.30	5-401.42	5-448.21
1-570.0	1-586.4	1-587.3	5-401.31	5-401.4x	5-448.2x
1-570.1	1-586.5	1-587.4	5-401.3x	5-401.h	5-448.x0

5-448.xx	5-462.7	5-541.2	5-651.a0	5-653	5-656.y
5-448.y	5-462.x	5-541.3	5-651.a3	5-653.20	5-659.20
5-449.4x	5-462.y	5-541.4	5-651.a4	5-653.23	5-659.23
5-449.70	5-467.x	5-545.0	5-651.ax	5-653.30	5-659.x0
5-449.k0	5-467.x0	5-545.1	5-651.b	5-653.33	5-659.x3
5-449.n0	5-467.x1	5-545.x	5-651.b0	5-653.3x	5-660.2
5-449.q	5-467.x2	5-545.y	5-651.b3	5-656	5-660.5
5-449.x0	5-467.x3	5-546	5-651.b4	5-656.8	5-661.40
5-450	5-467.xx	5-546.2	5-651.bx	5-656.80	5-661.43
5-450.0	5-467.y	5-546.20	5-651.x	5-656.83	5-661.50
5-450.1	5-469.b0	5-546.21	5-651.x0	5-656.84	5-661.53
5-450.2	5-469.bx	5-546.22	5-651.x3	5-656.8x	5-661.60
5-450.x	5-469.x0	5-546.2x	5-651.x4	5-656.9	5-661.63
5-450.y	5-469.xx	5-546.3	5-651.xx	5-656.90	5-661.6x
5-451	5-469.y	5-650	5-651.y	5-656.93	5-665.40
5-451.40	5-514.00	5-650.2	5-652	5-656.94	5-665.43
5-451.42	5-514.0x	5-650.5	5-652.4	5-656.9x	5-665.53
5-451.4x	5-514.20	5-650.6	5-652.40	5-656.a	5-665.x0
5-451.x0	5-514.30	5-650.x	5-652.43	5-656.a0	5-665.x3
5-451.xx	5-514.40	5-650.y	5-652.44	5-656.a3	5-692.03
5-452	5-514.50	5-651	5-652.4x	5-656.a4	5-692.13
5-452.30	5-514.5x	5-651.8	5-652.5	5-656.ax	5-692.x3
5-452.32	5-514.b0	5-651.80	5-652.50	5-656.b	5-695.03
5-452.3x	5-514.bx	5-651.83	5-652.53	5-656.b0	5-695.10
5-462	5-514.g0	5-651.84	5-652.54	5-656.b3	5-695.13
5-462.0	5-514.k0	5-651.8x	5-652.5x	5-656.b4	5-695.20
5-462.1	5-514.p3	5-651.9	5-652.6	5-656.bx	5-695.30
5-462.2	5-517.40	5-651.90	5-652.60	5-656.x	
5-462.3	5-518.10	5-651.93	5-652.63	5-656.x0	
5-462.4	5-541	5-651.94	5-652.64	5-656.x3	
5-462.5	5-541.0	5-651.9x	5-652.6x	5-656.x4	
5-462.6	5-541.1	5-651.a	5-652.y	5-656.xx	

Kat 145

	5-071.40	5-072.02	5-072.x1	5-073.1x	5-073.x2
5-070	5-071.41	5-072.0x	5-072.x2	5-073.2	5-073.xx
5-070.2	5-071.42	5-072.1	5-072.xx	5-073.20	5-073.y
5-070.3	5-071.4x	5-072.10	5-072.y	5-073.21	5-347
5-070.4	5-071.x	5-072.11	5-073	5-073.22	5-347.0
5-070.x	5-071.x0	5-072.12	5-073.0	5-073.2x	5-347.1
5-070.y	5-071.x1	5-072.1x	5-073.00	5-073.4	5-347.3
5-071	5-071.x2	5-072.2	5-073.01	5-073.40	5-347.30
5-071.0	5-071.xx	5-072.20	5-073.02	5-073.41	5-347.31
5-071.00	5-071.y	5-072.21	5-073.0x	5-073.42	5-347.4
5-071.01	5-072	5-072.22	5-073.1	5-073.4x	5-347.40
5-071.02	5-072.0	5-072.2x	5-073.10	5-073.x	5-347.41
5-071.0x	5-072.00	5-072.x	5-073.11	5-073.x0	5-347.5
5-071.4	5-072.01	5-072.x0	5-073.12	5-073.x1	5-347.50

5-347.51
5-347.6
5-347.60
5-347.61
5-347.62
5-347.63
5-347.x
5-347.y
5-391
5-391.0
5-391.1
5-391.2
5-391.3
5-391.x
5-391.y
5-401.6
5-401.b
5-402.2
5-402.3
5-402.5
5-402.7
5-402.8
5-402.9
5-402.a
5-402.b
5-402.g
5-402.h
5-404.d
5-404.e
5-404.f
5-404.g
5-404.m
5-404.n
5-412
5-413
5-413.0
5-413.00
5-413.02
5-413.1
5-413.10
5-413.12
5-413.x
5-413.y
5-419
5-419.0
5-419.1
5-419.2
5-419.5
5-419.x
5-419.y
5-420.0
5-420.00
5-420.04
5-420.20
5-420.24
5-420.2x
5-420.x0
5-424.2
5-426.0
5-426.02
5-426.03
5-426.04
5-426.12
5-426.13
5-426.14
5-426.22
5-426.23
5-426.24
5-426.x2
5-426.x3
5-426.x4
5-427.04
5-432.2
5-433
5-433.0
5-433.1
5-434
5-434.0
5-434.1
5-434.2
5-434.3
5-434.30
5-434.32
5-434.4
5-434.40
5-434.42
5-434.5
5-434.50
5-434.52
5-434.6
5-434.60
5-434.62
5-434.x
5-434.y
5-435
5-435.0
5-435.1
5-435.2
5-435.x
5-435.y
5-436
5-436.0
5-436.01
5-436.02
5-436.03
5-436.04
5-436.05
5-436.0x
5-436.1
5-436.11
5-436.12
5-436.13
5-436.14
5-436.15
5-436.1x
5-436.2
5-436.21
5-436.22
5-436.23
5-436.24
5-436.25
5-436.2x
5-436.x
5-436.x1
5-436.x2
5-436.x3
5-436.x4
5-436.x5
5-436.xx
5-436.y
5-437
5-437.0
5-437.01
5-437.02
5-437.03
5-437.04
5-437.05
5-437.0x
5-437.1
5-437.11
5-437.12
5-437.13
5-437.14
5-437.15
5-437.1x
5-437.2
5-437.21
5-437.22
5-437.23
5-437.24
5-437.25
5-437.2x
5-437.3
5-437.31
5-437.32
5-437.33
5-437.34
5-437.35
5-437.3x
5-437.4
5-437.41
5-437.42
5-437.43
5-437.44
5-437.45
5-437.4x
5-437.5
5-437.51
5-437.52
5-437.53
5-437.54
5-437.55
5-437.5x
5-437.6
5-437.61
5-437.62
5-437.63
5-437.64
5-437.65
5-437.6x
5-437.x
5-437.x1
5-437.x2
5-437.x3
5-437.x4
5-437.x5
5-437.xx
5-437.y
5-438
5-438.0
5-438.01
5-438.02
5-438.03
5-438.04
5-438.05
5-438.0x
5-438.1
5-438.11
5-438.12
5-438.13
5-438.14
5-438.15
5-438.1x
5-438.2
5-438.21
5-438.22
5-438.23
5-438.24
5-438.25
5-438.2x
5-438.3
5-438.31
5-438.32
5-438.33
5-438.34
5-438.35
5-438.3x
5-438.x
5-438.x1
5-438.x2
5-438.x3
5-438.x4
5-438.x5
5-438.xx
5-438.y
5-439
5-440
5-440.0
5-440.1
5-440.2
5-440.x
5-440.y
5-441
5-441.0
5-441.1
5-441.2
5-441.x
5-441.y
5-442
5-442.0
5-442.1
5-442.2
5-442.3
5-442.4
5-442.5
5-442.6
5-442.x
5-442.y
5-443
5-443.0
5-443.1
5-443.2
5-443.3
5-443.4
5-443.5
5-443.6
5-443.x
5-443.y
5-444.12
5-444.21
5-444.4
5-444.5
5-445
5-445.0
5-445.00
5-445.02
5-445.1
5-445.10
5-445.12
5-445.2
5-445.20
5-445.22
5-445.3
5-445.30
5-445.32
5-445.4
5-445.40
5-445.42
5-445.5
5-445.50
5-445.52
5-445.6
5-445.60
5-445.62
5-445.7
5-445.70
5-445.72
5-445.x
5-445.x0
5-445.x2
5-445.y
5-447
5-447.0
5-447.1

5-447.2	5-455.64	5-455.bx	5-458.07	5-458.x2	5-485.2x
5-447.3	5-455.65	5-455.c	5-458.0x	5-458.x3	5-485.3
5-447.4	5-455.66	5-455.c1	5-458.1	5-458.x4	5-485.4
5-447.5	5-455.67	5-455.c2	5-458.11	5-458.x5	5-485.5
5-447.6	5-455.6x	5-455.c3	5-458.12	5-458.x6	5-485.x
5-447.7	5-455.7	5-455.c4	5-458.13	5-458.x7	5-485.y
5-447.8	5-455.71	5-455.c5	5-458.14	5-458.xx	5-486
5-447.80	5-455.72	5-455.c6	5-458.15	5-458.y	5-486.3
5-447.82	5-455.73	5-455.c7	5-458.16	5-467.71	5-486.4
5-447.9	5-455.74	5-455.cx	5-458.17	5-467.72	5-496.6
5-447.90	5-455.75	5-455.d	5-458.1x	5-467.73	5-496.9
5-447.92	5-455.76	5-455.d1	5-458.2	5-467.7x	5-496.90
5-447.a	5-455.77	5-455.d2	5-458.21	5-467.80	5-496.91
5-447.a0	5-455.7x	5-455.d3	5-458.22	5-467.81	5-496.9x
5-447.a2	5-455.8	5-455.d4	5-458.23	5-467.82	5-500
5-447.b	5-455.81	5-455.d5	5-458.24	5-467.83	5-500.x
5-447.b0	5-455.82	5-455.d6	5-458.25	5-467.a	5-500.y
5-447.b2	5-455.83	5-455.d7	5-458.26	5-467.a0	5-501
5-447.c	5-455.84	5-455.dx	5-458.27	5-467.a1	5-501.0
5-447.c0	5-455.85	5-455.x	5-458.2x	5-467.ax	5-501.00
5-447.c2	5-455.86	5-455.x1	5-458.3	5-467.b0	5-501.02
5-447.x	5-455.87	5-455.x2	5-458.31	5-467.b1	5-501.03
5-447.y	5-455.8x	5-455.x3	5-458.32	5-467.b2	5-501.0x
5-449.40	5-455.9	5-455.x4	5-458.33	5-467.b3	5-501.1
5-449.42	5-455.91	5-455.x5	5-458.34	5-467.b4	5-501.10
5-449.50	5-455.92	5-455.x6	5-458.35	5-467.b5	5-501.12
5-449.52	5-455.93	5-455.x7	5-458.36	5-467.b6	5-501.13
5-455.4	5-455.94	5-455.xx	5-458.37	5-467.b7	5-501.1x
5-455.41	5-455.95	5-455.y	5-458.3x	5-467.b8	5-501.2
5-455.42	5-455.96	5-456	5-458.4	5-467.b9	5-501.20
5-455.43	5-455.97	5-456.0	5-458.41	5-467.ba	5-501.22
5-455.44	5-455.9x	5-456.00	5-458.42	5-467.bb	5-501.23
5-455.45	5-455.a	5-456.1	5-458.43	5-467.bc	5-501.2x
5-455.46	5-455.a1	5-456.16	5-458.44	5-467.bd	5-501.4
5-455.47	5-455.a2	5-456.2	5-458.45	5-467.be	5-501.40
5-455.4x	5-455.a3	5-456.21	5-458.46	5-469.80	5-501.42
5-455.5	5-455.a4	5-456.27	5-458.47	5-469.82	5-501.43
5-455.51	5-455.a5	5-456.2x	5-458.4x	5-469.g0	5-501.4x
5-455.52	5-455.a6	5-456.x	5-458.5	5-469.g2	5-501.5
5-455.53	5-455.a7	5-456.x8	5-458.51	5-482.b1	5-501.50
5-455.54	5-455.ax	5-456.y	5-458.52	5-485	5-501.52
5-455.55	5-455.b	5-458	5-458.53	5-485.0	5-501.53
5-455.56	5-455.b1	5-458.0	5-458.54	5-485.01	5-501.5x
5-455.57	5-455.b2	5-458.01	5-458.55	5-485.02	5-501.6
5-455.5x	5-455.b3	5-458.02	5-458.56	5-485.0x	5-501.60
5-455.6	5-455.b4	5-458.03	5-458.57	5-485.1	5-501.62
5-455.61	5-455.b5	5-458.04	5-458.5x	5-485.2	5-501.63
5-455.62	5-455.b6	5-458.05	5-458.x	5-485.21	5-501.6x
5-455.63	5-455.b7	5-458.06	5-458.x1	5-485.22	5-501.7

5-501.70
5-501.72
5-501.73
5-501.7x
5-501.x
5-501.x0
5-501.x2
5-501.x3
5-501.xx
5-501.y
5-502
5-502.0
5-502.1
5-502.2
5-502.3
5-502.4
5-502.5
5-502.6
5-502.7
5-502.8
5-502.x
5-502.y
5-503
5-503.0
5-503.1
5-503.2
5-503.3
5-503.4
5-503.5
5-503.6
5-503.x
5-503.y
5-505
5-505.0
5-505.1
5-505.2
5-505.x
5-505.y
5-509
5-509.0
5-509.00
5-509.0x
5-509.x
5-512
5-512.0
5-512.00
5-512.02
5-512.0x
5-512.1
5-512.10
5-512.12
5-512.1x
5-512.2
5-512.20
5-512.22
5-512.2x
5-512.3
5-512.30
5-512.32
5-512.3x
5-512.4
5-512.40
5-512.42
5-512.4x
5-512.x
5-512.x0
5-512.x2
5-512.xx
5-512.y
5-515
5-515.0
5-515.1
5-515.2
5-515.x
5-515.y
5-516
5-516.0
5-516.1
5-516.x
5-516.y
5-518.00
5-518.20
5-518.2x
5-518.40
5-519
5-519.0
5-519.1
5-519.2
5-519.3
5-519.4
5-519.x
5-519.y
5-520
5-520.0
5-520.1
5-520.2
5-520.x
5-520.y
5-521
5-521.0
5-521.1
5-521.2
5-521.3
5-521.4
5-521.40
5-521.4x
5-521.x
5-521.y
5-522
5-523
5-523.0
5-523.1
5-523.2
5-523.x
5-523.y
5-524
5-524.0
5-524.00
5-524.02
5-524.1
5-524.2
5-524.3
5-524.4
5-524.x
5-524.y
5-525
5-525.0
5-525.1
5-525.2
5-525.3
5-525.4
5-525.x
5-525.y
5-527
5-527.0
5-527.1
5-527.2
5-527.3
5-527.x
5-527.y
5-529
5-529.0
5-529.00
5-529.02
5-529.0x
5-529.1
5-529.10
5-529.12
5-529.1x
5-529.2
5-529.20
5-529.22
5-529.2x
5-529.3
5-529.30
5-529.32
5-529.3x
5-529.4
5-529.40
5-529.42
5-529.4x
5-529.5
5-529.50
5-529.52
5-529.5x
5-529.a
5-529.a0
5-529.a2
5-529.ax
5-529.b
5-529.b0
5-529.b2
5-529.bx
5-529.c
5-529.c0
5-529.c2
5-529.cx
5-529.d
5-529.d0
5-529.d2
5-529.dx
5-529.e
5-529.e0
5-529.e2
5-529.ex
5-529.f
5-529.f0
5-529.f2
5-529.fx
5-529.g
5-529.g0
5-529.g2
5-529.gx
5-529.h
5-529.h0
5-529.h2
5-529.hx
5-529.j
5-529.j0
5-529.j2
5-529.jx
5-529.k
5-529.k0
5-529.k2
5-529.kx
5-529.m
5-529.m0
5-529.m2
5-529.mx
5-529.n
5-529.n0
5-529.nx
5-529.q
5-529.q0
5-529.qx
5-529.r
5-529.r0
5-529.rx
5-529.x
5-529.x0
5-529.x2
5-529.xx
5-529.y
5-547.0
5-547.1
5-549.1
5-590
5-590.0
5-590.00
5-590.01
5-590.02
5-590.04
5-590.0x
5-590.1
5-590.10
5-590.11
5-590.12
5-590.1x
5-590.2
5-590.20
5-590.21
5-590.22
5-590.3
5-590.30
5-590.31
5-590.32
5-590.3x
5-590.4
5-590.40
5-590.41
5-590.42
5-590.4x
5-590.5
5-590.50
5-590.51
5-590.52
5-590.55
5-590.5x
5-590.60
5-590.61
5-590.62
5-590.71
5-590.8
5-590.80
5-590.81
5-590.82
5-590.84
5-590.85
5-590.8x
5-590.x
5-590.x0
5-590.x1
5-590.x2
5-590.x4
5-590.xx
5-590.y
5-603.0
5-603.00
5-603.10
5-604.01
5-604.02
5-604.11
5-604.12
5-604.x
5-605
5-609.1
5-609.2
5-681.30
5-681.32
5-681.34
5-681.80
5-681.82
5-681.84
5-681.92

5-681.94	5-682.13	5-682.x3	5-683.10	5-683.23	5-685.00
5-682.00	5-682.20	5-683.0	5-683.12	5-683.24	5-685.1
5-682.02	5-682.21	5-683.00	5-683.13	5-683.x0	5-685.42
5-682.03	5-682.22	5-683.03	5-683.14	5-683.x3	5-692.00
5-682.12	5-682.x2	5-683.04	5-683.20	5-683.x4	

Kat 146

5-530	5-530.1	5-530.33	5-530.6	5-530.74	5-530.9x
5-530.0	5-530.2	5-530.34	5-530.7	5-530.7x	5-530.x
5-530.00	5-530.3	5-530.3x	5-530.70	5-530.8	5-530.y
5-530.01	5-530.30	5-530.4	5-530.71	5-530.9	5-531.72
5-530.03	5-530.31	5-530.5	5-530.72	5-530.90	
5-530.0x	5-530.32	5-530.50	5-530.73	5-530.91	

Kat 147

5-534	5-534.34	5-535.2	5-535.x	5-536.44	5-536.x
5-534.0	5-534.35	5-535.3	5-535.y	5-536.45	5-536.y
5-534.01	5-534.36	5-535.30	5-536	5-536.46	5-539
5-534.02	5-534.37	5-535.31	5-536.0	5-536.47	5-539.0
5-534.03	5-534.38	5-535.33	5-536.1	5-536.48	5-539.1
5-534.0x	5-534.39	5-535.34	5-536.10	5-536.4a	5-539.2
5-534.1	5-534.3x	5-535.35	5-536.11	5-536.4b	5-539.30
5-534.2	5-534.x	5-535.36	5-536.1x	5-536.4c	5-539.31
5-534.3	5-534.y	5-535.37	5-536.2	5-536.4d	5-539.32
5-534.30	5-535	5-535.38	5-536.3	5-536.4f	5-539.4
5-534.31	5-535.0	5-535.39	5-536.41	5-536.4g	5-539.x
5-534.33	5-535.1	5-535.3x	5-536.43	5-536.4x	5-539.y

Kat 151

1-560	1-564.0	5-571	5-578.80	5-612.1	5-622.3
1-560.0	1-564.1	5-571.0	5-578.81	5-612.2	5-622.5
1-560.1	1-564.y	5-571.1	5-578.82	5-612.x	5-622.6
1-561	1-565	5-571.2	5-578.8x	5-612.y	5-622.x
1-561.0	1-569	5-571.3	5-581	5-613	5-622.y
1-561.1	1-569.0	5-571.x	5-581.0	5-613.0	5-624
1-562	1-569.1	5-571.y	5-581.1	5-613.1	5-625.y
1-562.0	1-569.2	5-572	5-581.x	5-613.2	5-627
1-562.1	1-569.3	5-572.0	5-581.y	5-613.x	5-627.2
1-562.2	1-569.y	5-572.1	5-610.0	5-613.y	5-627.x
1-562.3	5-550.1	5-572.2	5-610.1	5-619	5-627.y
1-562.x	5-550.6	5-572.3	5-610.2	5-620	5-628
1-562.y	5-570.1	5-572.4	5-610.x	5-621	5-628.2
1-563	5-570.2	5-572.5	5-610.y	5-622	5-628.3
1-563.0	5-570.3	5-572.x	5-611	5-622.0	5-628.4
1-563.1	5-570.x	5-572.y	5-612	5-622.1	5-628.x
1-564	5-570.y	5-578.8	5-612.0	5-622.2	5-628.y

5-629	5-630.1	5-630.y	5-633.0	5-634.2	5-640.2
5-629.0	5-630.2	5-631	5-633.1	5-634.x	5-640.4
5-629.x	5-630.3	5-631.0	5-633.x	5-634.y	5-641.0
5-629.y	5-630.4	5-631.1	5-634	5-639.0	5-643.0
5-630	5-630.5	5-631.x	5-634.0	5-640	
5-630.0	5-630.x	5-631.y	5-634.1	5-640.1	

Kat 152

5-530.02	5-624.4	5-625	5-625.x	5-626.2
5-530.51	5-624.5	5-625.4	5-626	5-626.3
5-622.7	5-624.x	5-625.5	5-626.0	5-626.x
5-622.8	5-624.y	5-625.6	5-626.1	5-626.y

Kat 153

	1-665	1-693.1	5-561.5	5-573.2	5-585
1-460	1-666	1-693.2	5-561.6	5-573.20	5-585.0
1-460.1	1-666.0	1-693.x	5-561.7	5-573.3	5-585.1
1-460.2	1-666.1	1-693.y	5-562.2	5-573.32	5-585.2
1-460.3	1-666.2	5-550.21	5-562.4	5-573.3x	5-585.3
1-460.4	1-666.x	5-550.31	5-562.5	5-573.40	5-585.x
1-460.5	1-666.y	5-552.2	5-562.8	5-573.41	5-585.y
1-462.2	1-668	5-552.6	5-569.62	5-573.x	5-596.00
1-462.3	1-668.0	5-560.0	5-569.72	5-573.y	5-596.01
1-660	1-668.1	5-560.1	5-569.x2	5-579.02	5-596.0x
1-661	1-668.2	5-560.2	5-570.0	5-579.04	8-100.b
1-663	1-668.x	5-560.30	5-570.4	5-579.14	8-100.c
1-663.0	1-668.y	5-560.3x	5-573	5-579.x2	8-137.00
1-663.1	1-693	5-560.8	5-573.0	5-582.1	8-137.10
1-663.y	1-693.0	5-561.2	5-573.1	5-582.3	8-137.20

Kat 154

5-550	5-551.2	5-552.5	5-553.03	5-553.x0	5-554.51
5-550.0	5-551.3	5-552.51	5-553.0x	5-553.x1	5-554.52
5-550.2	5-551.4	5-552.52	5-553.1	5-553.x2	5-554.53
5-550.20	5-551.5	5-552.53	5-553.10	5-553.x3	5-554.5x
5-550.2x	5-551.6	5-552.5x	5-553.11	5-553.xx	5-554.6
5-550.3	5-551.x	5-552.7	5-553.12	5-553.y	5-554.60
5-550.30	5-551.y	5-552.70	5-553.13	5-554	5-554.61
5-550.3x	5-552	5-552.7x	5-553.1x	5-554.4	5-554.62
5-550.4	5-552.0	5-552.x	5-553.2	5-554.40	5-554.63
5-550.5	5-552.1	5-552.y	5-553.20	5-554.41	5-554.6x
5-550.x	5-552.3	5-553	5-553.21	5-554.42	5-554.7
5-550.y	5-552.4	5-553.0	5-553.22	5-554.43	5-554.70
5-551	5-552.40	5-553.00	5-553.23	5-554.4x	5-554.71
5-551.0	5-552.41	5-553.01	5-553.2x	5-554.5	5-554.72
5-551.1	5-552.4x	5-553.02	5-553.x	5-554.50	5-554.73

5-554.7x	5-557.23	5-557.ax	5-563.x1	5-565.1x	5-566.81
5-554.8	5-557.2x	5-557.x	5-563.x2	5-565.2	5-566.8x
5-554.80	5-557.3	5-557.x0	5-563.xx	5-565.20	5-566.9
5-554.81	5-557.30	5-557.x1	5-563.y	5-565.21	5-566.90
5-554.82	5-557.31	5-557.x2	5-564	5-565.2x	5-566.91
5-554.83	5-557.32	5-557.x3	5-564.2	5-565.3	5-566.9x
5-554.8x	5-557.33	5-557.xx	5-564.20	5-565.30	5-566.a
5-554.9	5-557.3x	5-557.y	5-564.21	5-565.31	5-566.a0
5-554.90	5-557.4	5-559	5-564.22	5-565.3x	5-566.a1
5-554.91	5-557.40	5-559.0	5-564.2x	5-565.4	5-566.ax
5-554.92	5-557.41	5-559.1	5-564.3	5-565.40	5-566.b
5-554.93	5-557.42	5-559.10	5-564.30	5-565.41	5-566.b0
5-554.9x	5-557.43	5-559.12	5-564.31	5-565.4x	5-566.b1
5-554.a	5-557.4x	5-559.1x	5-564.32	5-565.5	5-566.bx
5-554.a0	5-557.5	5-559.2	5-564.3x	5-565.50	5-566.c
5-554.a1	5-557.50	5-559.3	5-564.4	5-565.51	5-566.c0
5-554.a2	5-557.51	5-559.32	5-564.40	5-565.5x	5-566.c1
5-554.a3	5-557.52	5-559.x	5-564.41	5-565.x	5-566.cx
5-554.ax	5-557.53	5-559.x0	5-564.42	5-565.x0	5-566.x
5-554.b	5-557.5x	5-559.x1	5-564.4x	5-565.x1	5-566.x0
5-554.b0	5-557.6	5-559.x2	5-564.5	5-565.xx	5-566.x1
5-554.b1	5-557.60	5-559.x3	5-564.50	5-565.y	5-566.xx
5-554.b2	5-557.61	5-559.xx	5-564.51	5-566	5-566.y
5-554.b3	5-557.62	5-559.y	5-564.52	5-566.0	5-567
5-554.bx	5-557.63	5-561.0	5-564.5x	5-566.00	5-567.0
5-554.x	5-557.6x	5-561.3	5-564.6	5-566.01	5-567.05
5-554.x0	5-557.7	5-561.8	5-564.60	5-566.0x	5-567.06
5-554.x1	5-557.70	5-562.0	5-564.61	5-566.1	5-567.1
5-554.x2	5-557.71	5-562.1	5-564.62	5-566.10	5-567.2
5-554.x3	5-557.72	5-563	5-564.6x	5-566.11	5-567.3
5-554.xx	5-557.73	5-563.0	5-564.7	5-566.1x	5-567.4
5-554.y	5-557.7x	5-563.00	5-564.70	5-566.2	5-567.7
5-557	5-557.8	5-563.01	5-564.71	5-566.20	5-567.8
5-557.0	5-557.80	5-563.02	5-564.72	5-566.21	5-567.x
5-557.00	5-557.81	5-563.0x	5-564.7x	5-566.2x	5-567.y
5-557.01	5-557.82	5-563.1	5-564.x	5-566.3	5-568.1
5-557.02	5-557.83	5-563.10	5-564.x0	5-566.30	5-568.1x
5-557.03	5-557.8x	5-563.11	5-564.x1	5-566.31	5-568.2
5-557.0x	5-557.9	5-563.12	5-564.x2	5-566.3x	5-568.20
5-557.1	5-557.90	5-563.1x	5-564.xx	5-566.4	5-568.21
5-557.10	5-557.91	5-563.2	5-564.y	5-566.40	5-568.2x
5-557.11	5-557.92	5-563.20	5-565	5-566.41	5-568.3
5-557.12	5-557.93	5-563.21	5-565.0	5-566.4x	5-568.30
5-557.13	5-557.9x	5-563.22	5-565.00	5-566.5	5-568.31
5-557.1x	5-557.a	5-563.2x	5-565.01	5-566.50	5-568.3x
5-557.2	5-557.a0	5-563.3	5-565.0x	5-566.51	5-568.8
5-557.20	5-557.a1	5-563.4	5-565.1	5-566.5x	5-568.80
5-557.21	5-557.a2	5-563.x	5-565.10	5-566.8	5-568.81
5-557.22	5-557.a3	5-563.x0	5-565.11	5-566.80	5-568.8x

5-568.9
5-568.90
5-568.91
5-568.9x
5-568.a
5-568.a0
5-568.a1
5-568.ax
5-568.b
5-568.b0
5-568.b1
5-568.bx
5-568.c
5-568.c0
5-568.c1
5-568.cx
5-568.d
5-568.d0
5-568.d1
5-568.dx
5-568.e
5-568.e0
5-568.e1
5-568.ex
5-568.f
5-568.g
5-568.g0
5-568.g1
5-568.gx
5-568.h
5-568.j
5-568.x
5-568.x0
5-568.x1
5-568.xx
5-568.y
5-569
5-569.0
5-569.0x
5-569.1
5-569.12
5-569.1x
5-569.2
5-569.2x
5-569.3
5-569.4
5-569.42
5-569.5
5-569.50
5-569.51
5-569.52
5-569.5x
5-569.6
5-569.60
5-569.61
5-569.6x
5-569.7
5-569.70
5-569.71
5-569.7x
5-569.x
5-569.x0
5-569.x1
5-569.xx
5-569.y
5-574
5-574.0
5-574.1
5-574.2
5-574.3
5-574.4
5-574.x
5-574.y
5-575
5-575.0
5-575.00
5-575.01
5-575.02
5-575.0x
5-575.2
5-575.20
5-575.21
5-575.2x
5-575.3
5-575.30
5-575.31
5-575.3x
5-575.4
5-575.40
5-575.41
5-575.4x
5-575.6
5-575.60
5-575.61
5-575.6x
5-575.7
5-575.70
5-575.71
5-575.7x
5-575.8
5-575.80
5-575.81
5-575.8x
5-575.9
5-575.90
5-575.91
5-575.9x
5-575.x
5-575.x0
5-575.x1
5-575.xx
5-575.y
5-576
5-576.0
5-576.00
5-576.01
5-576.0x
5-576.1
5-576.10
5-576.11
5-576.1x
5-576.2
5-576.20
5-576.21
5-576.2x
5-576.3
5-576.30
5-576.31
5-576.3x
5-576.4
5-576.40
5-576.41
5-576.4x
5-576.5
5-576.50
5-576.51
5-576.5x
5-576.6
5-576.60
5-576.61
5-576.6x
5-576.7
5-576.70
5-576.71
5-576.7x
5-576.8
5-576.80
5-576.81
5-576.8x
5-576.x
5-576.x0
5-576.x1
5-576.xx
5-576.y
5-577
5-577.0
5-577.00
5-577.01
5-577.0x
5-577.1
5-577.10
5-577.11
5-577.1x
5-577.2
5-577.20
5-577.21
5-577.2x
5-577.3
5-577.30
5-577.31
5-577.3x
5-577.4
5-577.40
5-577.41
5-577.4x
5-577.x
5-577.x0
5-577.x1
5-577.xx
5-577.y
5-578
5-578.0
5-578.00
5-578.01
5-578.02
5-578.0x
5-578.1
5-578.10
5-578.11
5-578.1x
5-578.2
5-578.20
5-578.21
5-578.2x
5-578.3
5-578.30
5-578.31
5-578.3x
5-578.4
5-578.40
5-578.41
5-578.4x
5-578.5
5-578.50
5-578.51
5-578.5x
5-578.6
5-578.60
5-578.61
5-578.62
5-578.6x
5-578.7
5-578.70
5-578.71
5-578.7x
5-578.x
5-578.x0
5-578.x1
5-578.xx
5-578.y
5-579
5-579.00
5-579.10
5-579.11
5-579.30
5-579.x1
5-580
5-580.0
5-580.1
5-582
5-582.0
5-582.2
5-582.4
5-582.x
5-582.y
5-583
5-583.0
5-583.1
5-583.2
5-583.3
5-583.x
5-583.y
5-584
5-584.0
5-584.1
5-584.2
5-584.3
5-584.4
5-584.5
5-584.6
5-584.7
5-584.70
5-584.71
5-584.72
5-584.73
5-584.74
5-584.7x
5-584.8
5-584.80
5-584.81
5-584.82
5-584.83
5-584.84
5-584.8x
5-584.9
5-584.a
5-584.x
5-584.y
5-593
5-593.0
5-593.00
5-593.01
5-593.02
5-593.0x
5-593.1
5-593.10
5-593.11
5-593.1x
5-593.2
5-593.20
5-593.2x
5-593.x
5-593.y
5-594
5-594.0
5-594.1
5-594.2
5-594.3
5-594.30
5-594.31
5-594.x
5-594.y
5-595
5-595.0

5-595.1
5-595.10
5-595.11
5-595.1x
5-595.2
5-595.20
5-595.21
5-595.22
5-595.23
5-595.24
5-595.25
5-595.2x
5-595.3
5-595.x
5-595.y
5-596.1
5-596.2
5-596.3
5-596.4
5-596.5
5-596.70
5-596.71
5-596.x
5-597
5-597.0
5-597.00
5-597.01
5-597.02
5-597.0x
5-597.2
5-597.3
5-597.30
5-597.31
5-597.32
5-597.33
5-597.34
5-597.35
5-597.36
5-597.3x
5-597.4
5-597.x
5-597.y
5-598.0
5-599.02
5-612.3
5-642.0
5-642.1
5-643
5-643.1
5-643.2
5-643.3
5-643.4
5-643.x
5-643.y
5-644
5-644.2
5-644.20
5-644.21
5-644.22
5-644.23
5-644.2x
5-645
5-645.0
5-645.2
5-645.20
5-645.21
5-645.22
5-645.23
5-645.2x
5-645.x
5-645.y
5-646.0
5-646.1
5-649.2
5-649.4
5-649.9

Kat 181

5-37b
5-37b.0
5-37b.00
5-37b.01
5-37b.02
5-37b.1
5-37b.10
5-37b.11
5-37b.12
5-37b.21
5-399.4
5-399.5
5-506.0
5-506.
