# Supplementary figures and images for: Mind the Gap: A Nationwide Analysis of Case Distribution, Resident Exposure and Institutional Variation in German Pediatric Surgery Training
Source: Children (Basel). 2026 Apr 16;13(4):554. doi: 10.3390/children13040554 (PMC13114497; doi:10.3390/children13040554)

Pediatric Surgery in Germany 2012-2023

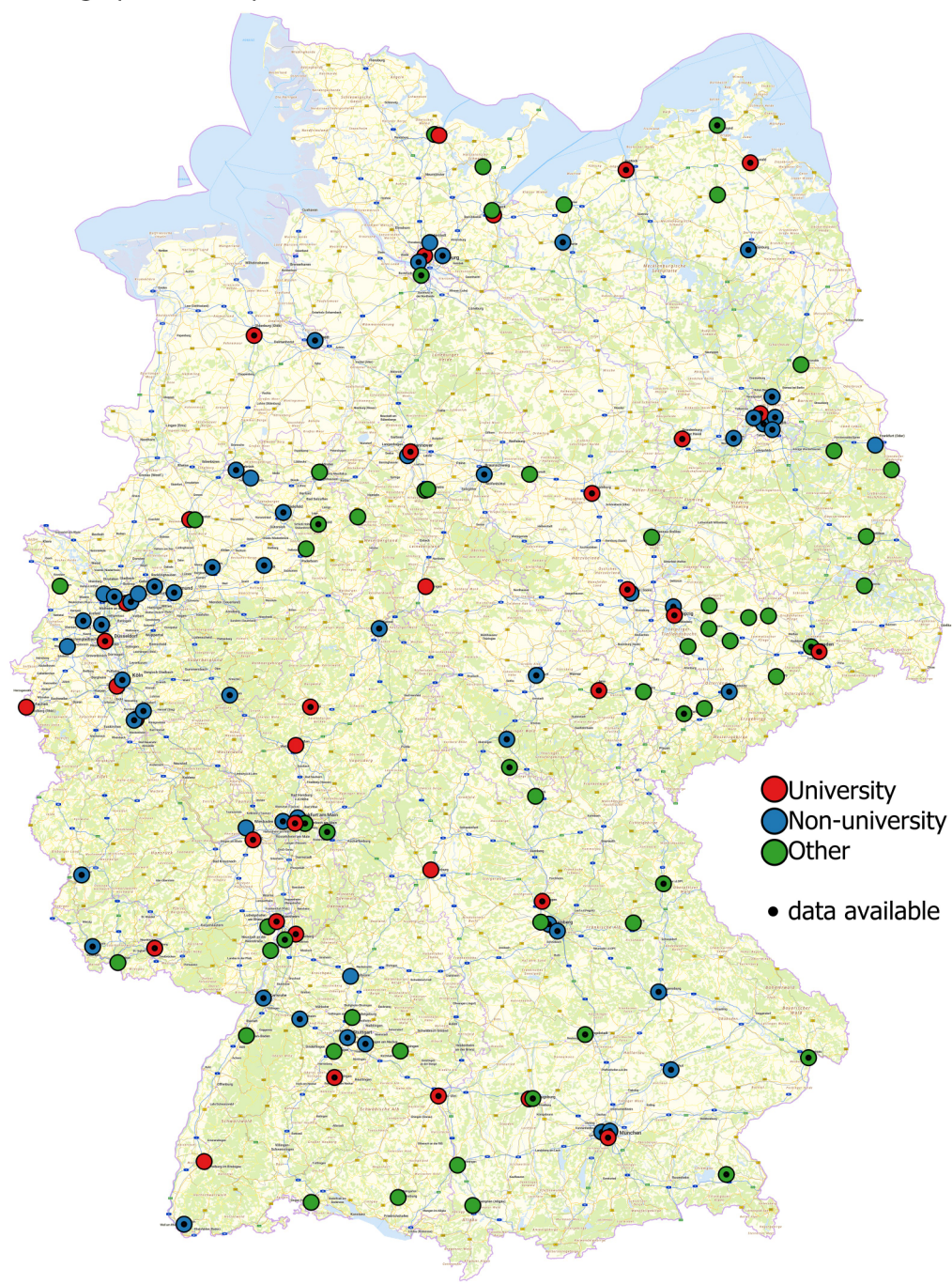

Supplement: Supplementary file 1 [file children-13-00554-s001.zip › Supplement 2 Map.pdf]
